# Role of Spin Polarization and Dynamic Correlation in Singlet–Triplet Gap Inversion of Heptazine Derivatives

**DOI:** 10.1021/acs.jctc.3c00781

**Published:** 2023-10-21

**Authors:** Daria Drwal, Mikulas Matousek, Pavlo Golub, Aleksandra Tucholska, Michał Hapka, Jiri Brabec, Libor Veis, Katarzyna Pernal

**Affiliations:** † Institute of Physics, Lodz University of Technology, ul. Wolczanska 219, 90-924 Lodz, Poland; ‡ J. Heyrovský Institute of Physical Chemistry, Academy of Sciences of the Czech Republic, v.v.i., Dolejškova 3, 18223 Prague 8, Czech Republic; § Faculty of Mathematics and Physics, Charles University, 12116 Prague, Czech Republic; ∥ Faculty of Chemistry, University of Warsaw, ul. L. Pasteura 1, 02-093 Warsaw, Poland

## Abstract

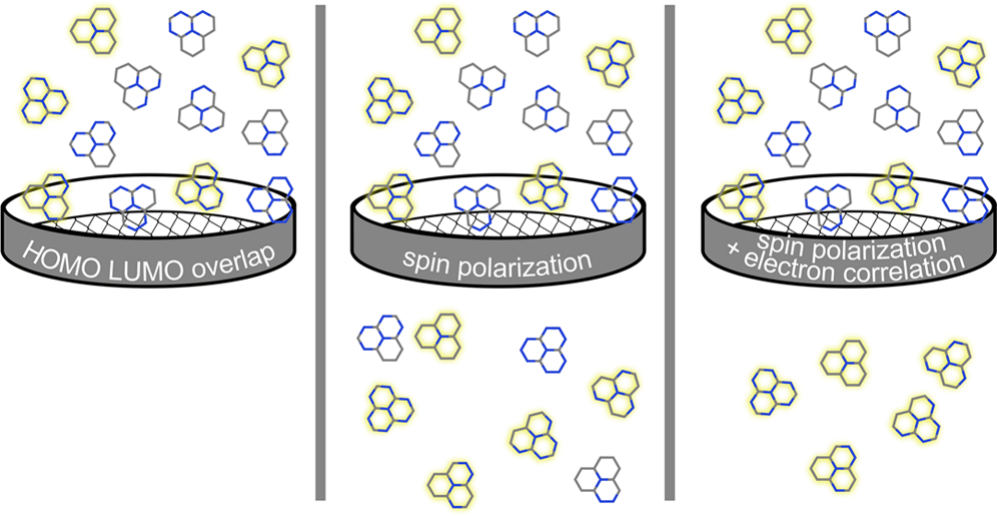

The new generation of proposed light-emitting molecules for organic light-emitting diodes (OLEDs) has raised considerable research interest due to its exceptional feature—a negative singlet–triplet (ST) gap violating Hund’s multiplicity rule in the excited S_1_ and T_1_ states. We investigate the role of spin polarization in the mechanism of ST gap inversion. Spin polarization is associated with doubly excited determinants of certain types, whose presence in the wave function expansion favors the energy of the singlet state more than that of the triplet. Using a perturbation theory-based model for spin polarization, we propose a simple descriptor for prescreening of candidate molecules with negative ST gaps and prove its usefulness for heptazine-type molecules. Numerical results show that the quantitative effect of spin polarization decreases linearly with the increasing highest occupied molecular orbital–lowest unoccupied molecular orbital (HOMO–LUMO) exchange integral. Comparison of single- and multireference coupled-cluster predictions of ST gaps shows that the former methods provide good accuracy by correctly balancing the effects of doubly excited determinants and dynamic correlation. We also show that accurate ST gaps may be obtained using a complete active space model supplemented with dynamic correlation from multireference adiabatic connection theory.

## Introduction

Detecting candidates for organic light-emitting diodes (OLEDs) through the screening of potential chromophores remains a challenge for quantum chemistry methods. OLED molecules may rely on different light-emitting mechanisms, ranging from pure fluorescence or phosphorescence (first- and second-generation emitters) to more complex processes aimed at harvesting nonfluorescent triplet excitons (third and fourth generations).^1,2^ Typically, organic molecules obey Hund’s multiplicity rule^3^ and the triplet state T_1_ is lower than the first excited singlet state S_1_. Hence, only a minor part of the excitons is available for photon emission through the radiative recombination of singlets. This restricts the internal quantum efficiency (IQE) to 25% when the distribution of excitons between S_1_ and T_1_ states is most favorable.^4^ For molecules characterized by small singlet–triplet gaps, it is possible to thermally induce reverse intersystem crossing (RISC), i.e., the transition from lower-lying T_1_ (dark state) to higher S_1_ (bright state). This process, known as thermally activated delayed fluorescence (TADF), results in higher-rate fluorescent emission from the singlet state.^5−9^

Recent attention has been focused on systems with inverted singlet–triplet gap (INVEST) molecules. In INVEST emitters, the S_1_ state lies below T_1_, so that the relaxation from T_1_ takes place with no need for thermal induction and 100% IQE of luminescence could, in principle, be achieved.^10−13^ It is worth mentioning that aromatic chromophores with negative S_1_–T_1_ energy difference (ST gap) are of interest in another area of active research, namely, in water-splitting photocatalysis. It has been shown that derivatives of heptazine may act as efficient photocatalysts. Thanks to the negative ST energy gap, the S_1_ state is exceptionally long-lived, as it does not suffer from quenching through intersystem crossing (ISC) to T_1_.^14^

The first calculations proving the possibility of ST gap inversion were carried out independently in 2019 by de Silva for cycl[3.3.3]azine^10^ and Sobolewski, Domcke, and co-workers for heptazine.^15^ Ref (15) also presented the first indirect experimental proof for the gap inversion and indicated that this phenomenon could explain the high efficiency of OLEDs observed already in 2013^16^ and 2014^17,18^ by Adachi and co-workers. A subsequent theoretical study by Sobolewski and Domcke^11^ confirmed this hypothesis. The works of Sancho-Garcia and co-workers^19,20^ demonstrated the ST gap inversion across N- and B-doped triangulenes of different sizes. In 2022, Aizawa and co-workers^21^ proposed a prescreening approach for INVEST candidates. Based on the elimination process, two heptazine analogues were chosen for further experimental evaluation, which confirmed the gap inversion.

The magnitude of the S_1_–T_1_ energy gap is directly related to the exchange integral involving the highest occupied molecular orbital (HOMO) and lowest unoccupied molecular orbital (LUMO).^22−24^ A vanishing overlap between frontier orbitals is a prerequisite for ST inversion but is not sufficient. Several studies have demonstrated that accounting for electron correlation via the inclusion of double excitations is essential to stabilize the singlet state with respect to triplet and obtain negative ST gaps of chromophore molecules.^10,12,24^ Results from single-reference response methods suggest that double excitations contribute at a relatively low level to the S_1_ state, estimated at ca. 10% by de Silva.^10^ Time-dependent density functional theory (TD-DFT) approaches are incapable of capturing double excitations and do not predict negative gaps, as corroborated by a range of investigations.^10,15,19,20,,26^

In pursuit of efficient strategies in designing novel INVEST materials, Pollice et al.^23^ investigated all possible permutations of cyclazine and heptazine derivatives with C–H replaced by a set of electron-donating and electron-withdrawing substituents and described them at the EOM-CCSD and TD-DFT levels of theory. Aizawa and co-workers^21^ expanded on this work by introducing 186 new substituents and finding almost 35,000 potential candidates for INVEST molecules. Their further TD-DFT screening resulted in a significant limitation of this set. Still, TD-DFT-based screening is inherently limited, as the method cannot distinguish INVEST molecules from cases in which the ST gap is small but not negative. Having studied the relationship between the structure and properties of selected INVEST molecules, Olivier and co-workers^13^ formulated a set of design rules in which the *C*_2*v*_ point group of the triangulene core was identified as a prerequisite for the gap inversion.

Advances in designing novel INVEST systems are hindered by two factors: a limited understanding of the mechanism behind gap reversal at the electronic structure level and the lack of efficient descriptors that could account for the main effects lowering S_1_ below T_1_. In one of the first theoretical works devoted to the violation of Hund’s rule in closed-shell molecules, Kollmar and Staemmler^22^ introduced a concept of dynamic spin polarization (sp), which associates the ST gap inversion to the energetic effect exerted by a small subset of doubly excited configurations involving frontier orbitals. Numerical investigations on conjugated hydrocarbons carried out by Koseki et al.^27^ showed that spin polarization may indeed lead to gap inversion in some molecules. Spin polarization has been also indirectly identified as a key effect in ST gap inversion of the heptazine molecule, without, however, providing its quantitative measure.^15^

The main objective of our study is to elucidate the role of both spin polarization and dynamic correlation energy in the mechanism of ST gap inversion in heptazine-based molecules. Based on the spin polarization model, we propose a simple descriptor for the prescreening of molecules with a negative ST gap. We also demonstrate the efficacy of selected multireference methods in accurately predicting the magnitudes of ST gaps in organic INVEST emitters. We compare multireference adiabatic connection methods^28−34^ and the second-order *n*-electron valence state perturbation theory (NEVPT2)^35^ against Mukherjee’s multireference coupled-cluster with noniterative triple excitations [Mk-MRCCSD(T)].^36^

## Computational Details

**Figure 1:**
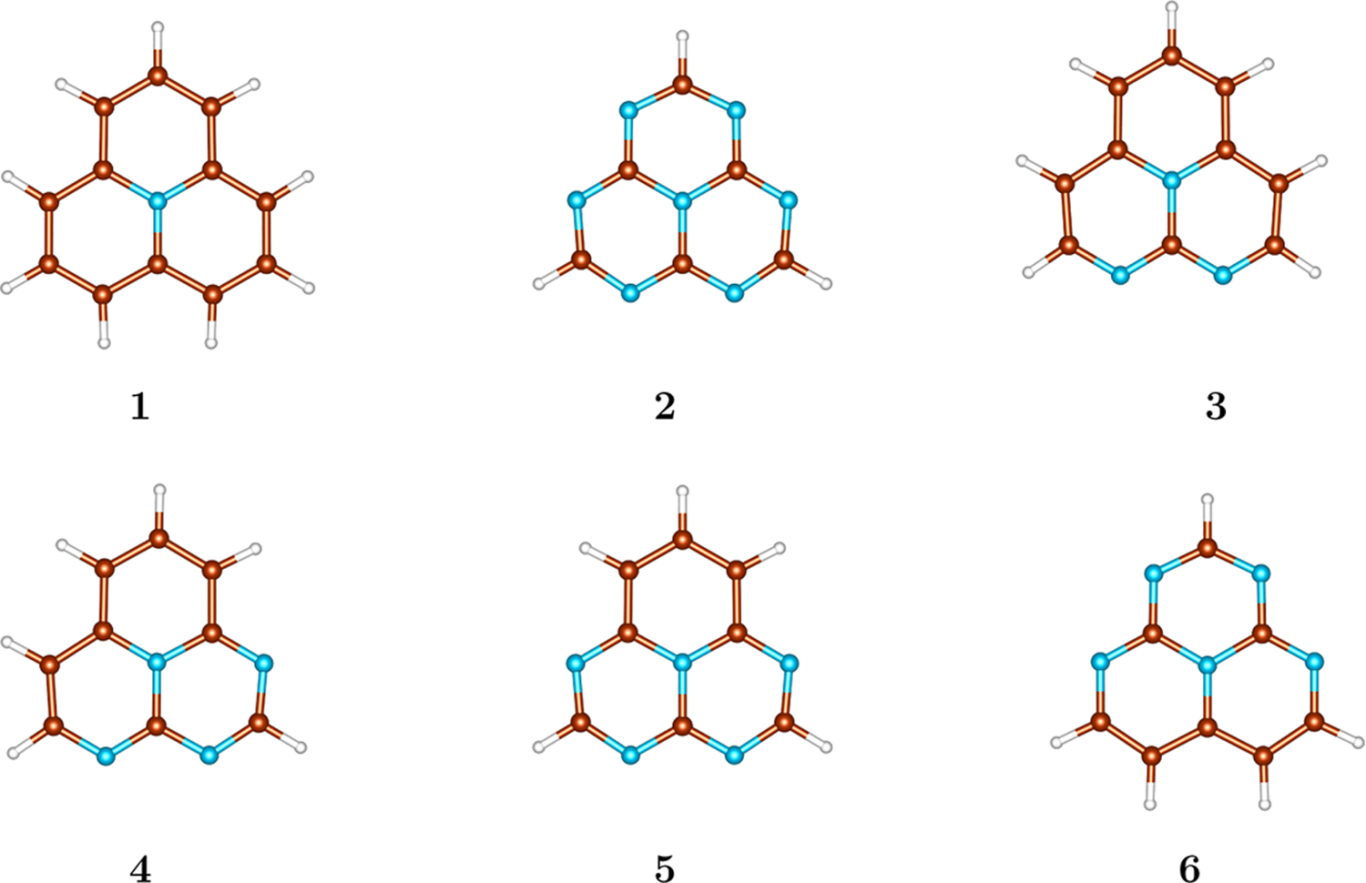
Structures of the studied molecules. The color codes are as follows: N (blue), C (brown), and H (white).

Our analysis is focused on six heptazine-based systems (see Figure 1). Geometries were optimized at the MP2 level using the Molpro^37^ program. All results were obtained using the def2-TZVP^38^ basis set.

Complete active space self-consistent field (CASSCF) and NEVPT2 results were obtained with the Molpro^37^ program. We used the same software for restricted Hartree–Fock (HF), CASSCF, and Kohn–Sham DFT calculations to obtain electron integrals and one- and two-electron-reduced density matrices needed for spin polarization models and adiabatic connection, AC0 and ACn, calculations. The latter were performed with the GammCor program.^39^ Multireference coupled-cluster Mk-MRCCSD(T)^36^ energies for the S_1_ and T_1_ states computed using the CASSCF(2,2) natural orbitals were obtained with the NWChem program^40^ and set as benchmark. CC2 results in def2-TZVP were taken from ref (24), while EOM-CCSD ST gaps were calculated with the Orca code.^41^

The ground state geometry of each molecule was used for both the S_1_ and T_1_ states. Except for system 4, calculations were performed by employing the *C*_2*v*_ point group symmetry, which allowed for state-specific CASSCF calculations for the S_1_ and T_1_ states. System 4 is in a lower, *C*_*s*_, point group symmetry, so that a two-state state-average CASSCF calculation is required to access the S_1_ state.

Additional calculations were performed on an extended test set including heptazine derivatives obtained by substituting C–H groups with N atoms in all possible ways. Geometries of all molecules from the extended set were optimized at the B3LYP/cc-pVDZ level with no symmetry constraints using Orca code.^41^

## Inverting S_1_–T_1_ Energy Gaps by Including Dynamic Spin Polarization

Throughout the text, Δ*E*_ST_ will denote the singlet–triplet energy gap defined as a difference of T_1_ energy subtracted from the energy of the S_1_ state, namely
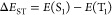
1Gap inversion would be equivalent to obtaining a negatively valued Δ*E*_ST_ energy difference.

In the first approximation, the S_1_ and T_1_ states of the considered systems can be described by singly excited determinants, where one electron from the HOMO (*H*) orbital is excited to the orbital LUMO (*L*)
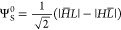
2
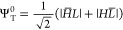
3A notation |*H̅L*| stands for an *N*-electron Slater determinant comprising *N*/2 – 1 doubly occupied orbitals and *H*, *L* singly occupied orbitals. *H̅*, *L̅* and *H*, *L* indicate orbitals with α and β spin components, respectively. In addition, we use {*pq*} and {*pqrs*} symbols to indicate which orbitals in open-shell determinants are singly occupied without explicitly specifying their spin components. For example, both determinants | *H̅L*| and |*H L̅*| would be denoted as {*HL*}.

As it is well-known, the ST energy gap corresponding to a wave function including only {*HL*} singly excited determinants (eqs 2 and 3)
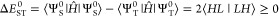
4is determined by the magnitude of the HOMO–LUMO (HL) exchange integral (throughout the text, two-electron integrals are written in the ⟨12|12⟩ convention) and is non-negative. eq 4 is a manifestation of Hund’s rule satisfied by wave functions Ψ_S_^0^ and Ψ_T_^0^.

In Hund’s picture, the ST gap is determined by the coupling of *H* and *L* electrons in the target spin state of the total wave function. This approach ignores the interaction of the unpaired α and β electrons with the core electrons. However, Kollmar and Staemmler^22^ showed that including these interactions is not merely a quantitative refinement, but may explain the ST gap inversion in some systems. Their approach is based on extending the first-order wave function with double excited determinants {*iaHL*}, where core orbitals *i* relax by excitations to an arbitrary virtual orbital *a*. Including such determinants is referred to as “dynamic” spin polarization to distinguish from the conventional “static” spin polarization. While static spin polarization leads to unequal spin components of electron density, dynamic spin polarization does not lead to nonzero spin density. Our goal is to quantify the effect of dynamic spin polarization for heptazine-type molecules and investigate if this mechanism is responsible for changing the sign of Δ*E*_ST_^0^ gaps. We also investigate if the sp-based gap inversion model of Kollmar and Staemmler can be employed for prescreening molecules likely to act as INVEST systems.

We begin by following ref (22) and construct singlet functions by applying *i* → *a* excitations to determinants in the Ψ_S_^0^ function, which leads to two functions
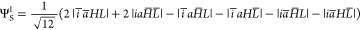
5
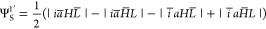
6Analogously, three triplet *M*_S_ = 0 functions can be generated from Ψ_T_^0^ by considering *i* → *a* excitations and they read
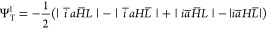
7
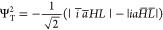
8
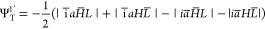
9Recall that from the Epstein–Nesbet perturbation theory (PT), the drop in the unperturbed energy, *E*^0^ = ⟨Ψ^0^|*Ĥ*|Ψ^0^⟩, that results from including in the wave function a configuration Ψ′ and corresponds to the second-order correction, is given by |⟨Ψ^0^|*Ĥ*|Ψ′⟩|^2^/(*E*^0^ – *E*′), where *E*′ = ⟨Ψ′|*Ĥ*|Ψ′⟩. After evaluating PT terms for functions given in eqs 5–9 (see Supporting Information for explicit expressions of Hamiltonian elements and energy differences in terms of two-electron integrals) and subtracting triplet contributions from the singlet ones, the following spin-free expression is obtained
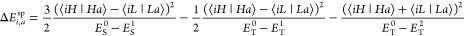
10in agreement with ref (22) (notice that contributions to Δ*E*_*i*,*a*_^sp^ from states Ψ_S_^1′^ and Ψ_T_^1′^, not considered in ref (22), cancel each other). Δ*E*_*i*,*a*_^sp^ represents a contribution to the ST gap from spin polarization of two electrons occupying orbital *i* by allowing their excitation to orbital *a*. To ease its interpretation, the expression in eq 10 can be approximated by assuming a common denominator given as Hartree–Fock orbital energy difference ε_*i*_ – ε_*a*_ for all three terms, leading to
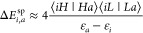
11It is now clear that spin polarization energy, Δ*E*_*i*,*a*_^sp^, pertaining to a pair of orbitals (*i*, *a*), where *i* < *H* and *a* > *L*, stabilizes the singlet state with respect to triplet if orbitals *i* and *a* overlap significantly with both *H* and *L* orbitals and, taking into account that ϵ_*a*_ – ϵ_*i*_ > 0, exchange integrals ⟨*iH*|*Ha*⟩ and ⟨*iL*|*La*⟩ are of the opposite sign. The magnitude of the Δ*E*_*i*,*a*_^sp^ energy is dependent on the closeness of the *i* and *a* orbital energy levels.

For the studied systems, the (*H* – 1, *H* – 2) and (*L* + 1, *L* + 2) pairs of orbitals are degenerate, so the simplest model for the ST gap accounting for major spin polarization would include two pairs of orbitals, and the ST energy gap expression of such a “12” model would read
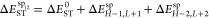
12For system 4, the model also included the contributions from the other two combinations of orbitals Δ*E*_*H*–1,*L*+2_^sp^ + Δ*E*_*H*–2,*L*+1_^sp^, which are negligible in the other systems due to point group symmetry. To fully account for spin polarization, considered pairs of orbitals (*i* < *H*, *a* > *L*) must fulfill conditions leading to ST gap reversal, formulated below eq 11: (i) orbitals within each pair should strongly overlap with *H* and *L* orbitals, (ii) the exchange integrals ⟨*iH*|*Ha*⟩ and ⟨*iL*|*La*⟩ are of the opposite signs. For heptazine derivatives, the π orbitals satisfy these requirements and the all-π sp-inclusive ST energy gap reads
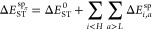
13(orbitals *i* and *a* are of the π-type).

**Figure 2:**
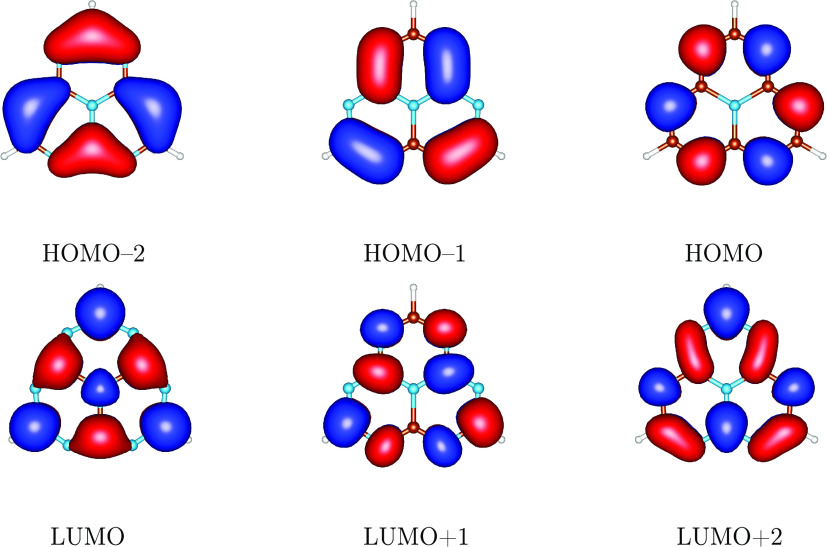
Natural orbitals of system 2 corresponding to state-averaged CASSCF(14,14) calculations with one singlet and one triplet states.

We consider two sets of orbitals in further analysis. The first set is given by canonical HF orbitals. The second one corresponds to a ground state CASSCF(14,14) wave function with the active space containing all the π electrons and orbitals (2p_*z*_ orbitals on carbon and nitrogen atoms plus one virtual orbital of the “double-shell” 3p_*z*_ character^24^). The CASSCF(14,14) natural occupation numbers for systems 2 and 4, used as illustrative cases, are given in Table 1. Notice that orbitals *H* and *L* pertain to (*N*/2)th and (*N*/2 + 1)th orbitals, respectively, of occupation numbers close to 0.5. These orbitals are depicted in Figure 2 (see also Figures 1–12 in Supporting Information) together with four other most strongly correlated, i.e., of the occupancies deviating most from 0 and 1, orbitals.

**Table 1:** Natural Occupation Numbers for Systems 2 and 4 from State-Averaged CASSCF(14,14) Calculations, for System 2 Corresponding to the Natural Orbitals Presented in Figure 2

	sys 2		sys 4	
orbital	S_1_	T_1_	S_1_	T_1_
*H* – 6	0.995	0.993	0.990	0.989
*H* – 5	0.969	0.970	0.971	0.971
*H* – 4	0.967	0.968	0.970	0.971
*H* – 3	0.967	0.968	0.969	0.971
*H* – 2	0.928	0.933	0.936	0.939
*H* – 1	0.929	0.933	0.933	0.936
*H*	0.513	0.504	0.639	0.509
*L*	0.485	0.492	0.359	0.486
*L* + 1	0.074	0.073	0.070	0.071
*L* + 2	0.074	0.073	0.069	0.070
*L* + 3	0.036	0.035	0.033	0.031
*L* + 4	0.036	0.035	0.032	0.031
*L* + 5	0.026	0.023	0.024	0.022
*L* + 6	0.001	0.001	0.004	0.004
CI Coefficients				
{*HL*}	68.6%	69.3%	67.3%	71.3%
{(*H* – *p*) (*L* + *p*) *H L*}_*p*=1,2_	8.2%	7.8%	7.2%	0.3%
{*i a H L*}_*i*,*a*∈π_	15.0%	14.6%	13.4%	5.6%

The results for ST gaps obtained without spin polarization, Δ*E*_ST_^0^, with partial and full spin polarization, Δ*E*_ST_^sp_12_^ and Δ*E*_ST_^sp_π_^ models, respectively, are presented in Table 2. First, it can be seen that Δ*E*_ST_^0^ gaps are positive and small, which indicates that for all considered systems the *H* and *L* orbitals overlap marginally at both the HF and CASSCF(14,14) levels of theory. Accounting for dynamic spin polarization from only two pairs of orbitals (*H* – 1, *L* + 1) and (*H* – 2, *L* + 2) (see eq 12) significantly reduces ST gaps. Using canonical HF orbitals leads to negative ST gaps for systems 1 and 2, while with CASSCF(14,14) natural orbitals, all ST gaps turn negative. Considering all (*i* < *H*, *a* > *L*) pairs of π orbitals (eq 13) removes another 0.2–0.3 eV from the ST gap, regardless of the employed orbitals. We conclude that the PT-based model for dynamic spin polarization substantially lowers the S_1_ state energy with respect to T_1_, possibly leading to a sign reversal of the ST gap. The combined sp effect of two pairs of orbitals, (*H* – 1, *L* + 1) and (*H* – 2, *L* + 2), accounts for more than half of the ST gap reduction.

**Table 2:** S_1_–T_1_ Energy Gaps Obtained without Spin Polarization, Δ*E*_ST_^0^, with Spin Polarization Computed from Two Pairs of π Orbitals, Δ*E*_ST_^sp_12_^, from All Occupied-Virtual Pairs of π Orbitals, Δ*E*_ST_^sp_π_^, and from CI with {*HL*}, {*H* – 1 *L* + 1 *H L*}, and {*H* – 2 *L* + 2 *H L*} Determinants, Δ*E*_ST_^CI_12_^^a^

orbitals	ST gap	1	2	3	4	5	6
HF	Δ*E*_ST_^0^	0.25	0.25	0.49	0.67	0.68	0.61
	Δ*E*_ST_^sp_12_^	–0.16	–0.18	0.09	0.29	0.33	0.22
	Δ*E*_ST_^sp_π_^	–0.37	–0.45	–0.11	0.09	0.07	0.00
	Δ*E*_ST_^CI_12_^	–0.21	–0.25	0.10	0.27	0.10	0.08
CASSCF	Δ*E*_ST_^0^	0.19	0.19	0.25	0.32	0.25	0.28
	Δ*E*_ST_^sp_12_^	–0.33	–0.40	–0.28	–0.19	–0.31	–0.27
	Δ*E*_ST_^sp_π_^	–0.60	–0.73	–0.54	–0.46	–0.61	–0.55
	Δ*E*_ST_^CI_12_^	–0.29	–0.36	–0.25	–0.14	–0.28	–0.24

a HF canonical and CASSCF(14,14) orbitals were used. The latter followed state-averaged CASSCF calculations with one singlet state and one triplet state. All values are in eV.

Spin polarization in the wave function picture is manifested by the presence of doubly excited determinants. To check if the simple PT-based model for sp yields qualitatively correct predictions, we have performed CI calculations constructing the S_1_ and T_1_ wave functions from {*HL*}, {*H* – 1 *L* + 1 *H L*}, and {*H* – 2 *L* + 2 *H L*} singly excited and doubly excited determinants, employing either HF or CASSCF(14,14) orbitals. The CI results, denoted as Δ*E*_ST_^CI_12_^ in Table 2, are in close agreement with those of the Δ*E*_ST_^sp_12_^ model. This suggests that the PT-based approach can be used as a cost-saving alternative to CI calculations in the prescreening for molecules with inverted gaps.

ST gaps obtained from Δ*E*_ST_^sp_12_^ and Δ*E*_ST_^sp_π_^ should be considered as basic approximations due to the fact that there are only {*HL*} and {*iaHL*} determinants in the wave functions. Thus, the model gaps cannot be quantitatively correct. A comparison with Mk-MRCCSD(T) results, shown in the last column of Table 3, reveals that ST gaps predicted by sp_12_ and sp_π_ models employed with HF orbitals (cf. Table 2) deviate on average by 0.26 and 0.15 eV from the Mk-MRCCSD(T) benchmarks, respectively. With CASSCF(14,14) orbitals, the respective mean absolute errors amount to 0.14 and 0.43 eV. Such inaccuracies result from the lack of dynamic electron correlation in the considered models. This problem is addressed in the next section.

**Table 3:** S_1_–T_1_ Energy Gaps in eV Obtained from CASSCF, AC_0_, AC*_n_*, and NEVPT2 Methods for Small, Intermediate, and All-π Models for Active Spaces Compared against CC2 and Mk-MRCCSD(T) Values

system	active space	CAS	AC_0_	AC*_n_*	NEVPT2	CC2^24^	Mk-MRCCSD(T)
1	(2,2)	1.76	0.70	0.00	–0.37	–0.13	–0.18
	(6,6)	–0.18	0.16	0.01	–0.08		
	(14,14)	–0.47	–0.10	–0.21	0.07		
2	(2,2)	0.31	–0.78	–0.28	–1.04	–0.24	–0.28
	(6,6)	–0.31	–0.02	–0.14	1.01		
	(14,14)	–0.62	–0.21	–0.34	–0.06		
3	(2,2)	0.44	–0.28	0.02	–0.59	–0.11	–0.15
	(6,6)	–0.17	0.08	–0.01	–0.04		
	(14,14)	–0.45	–0.05	–0.19	0.09		
4	(2,2)	0.79	–0.45	–0.22	–0.92	–0.08	–0.04
	(6,6)	0.18	–0.32	–0.01	–0.59		
	(14,14)	–0.31	–0.04	–0.12	–0.07^a^		
5	(2,2)	0.70	–0.64	–0.06	–0.94	–0.14	–0.15
	(6,6)	–0.12	0.04	–0.33	–0.25		
	(14,14)	–0.50	–0.06	–0.20	0.09		
6	(2,2)	0.72	–0.67	–0.06	–0.98	–0.12	–0.14
	(6,6)	–0.22	0.08	–0.08	0.05		
	(14,14)	–0.50	–0.01	–0.15	0.11		

a Due to problems with the convergence of NEVPT2, the active space has been reduced to (14,13).

**Figure 3:**
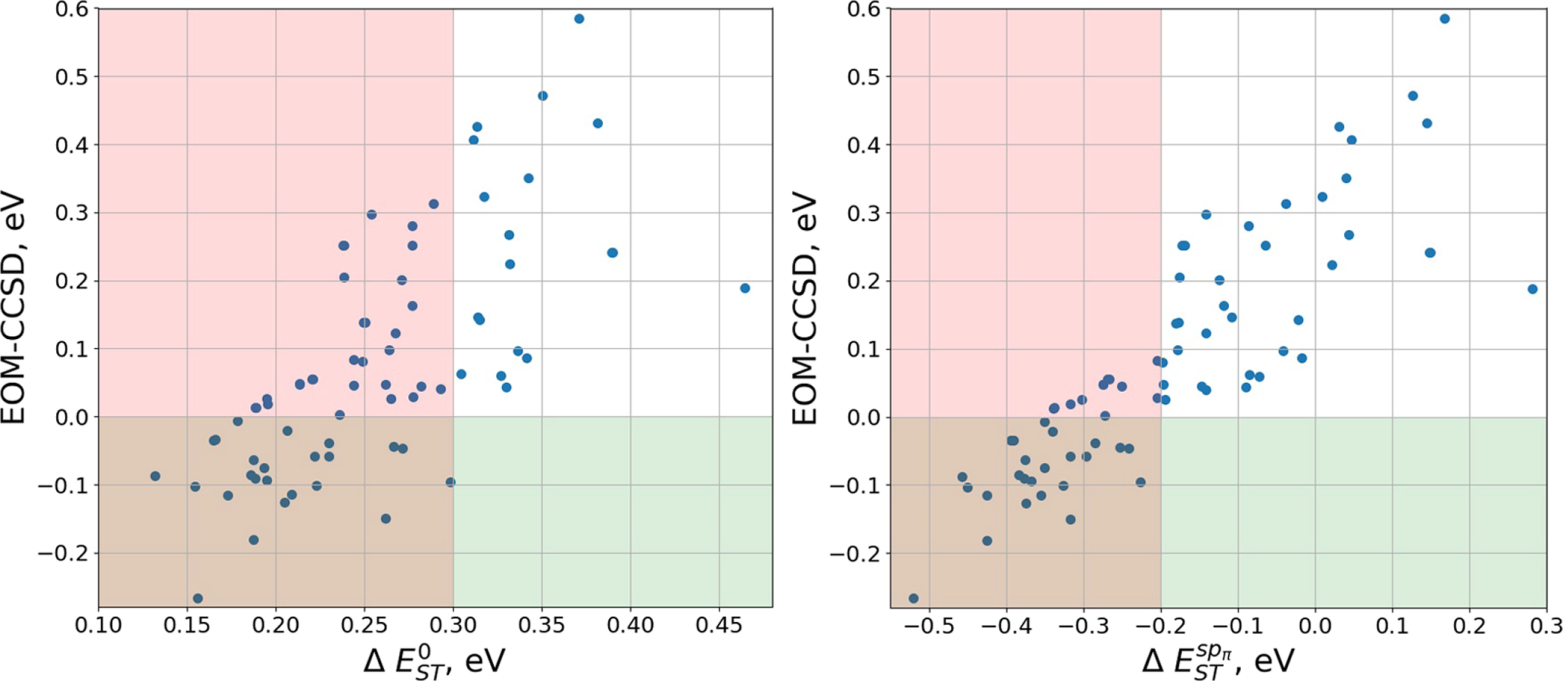
Left panel: ST energy gaps without spin polarization (eq 4). Right panel: ST energy gaps with spin polarization from all occupied-virtual π orbital pairs (eqs 13 and 10). Results obtained with BLYP ground state orbitals vs EOM-CCSD values for the extended test set of molecules.

To further explore if the sp effect is decisive in inverting the ST gap and if the model presented in eq 13 would be useful in screening for INVEST candidates, we have applied it to an extended test set of molecules including heptazine-derived systems obtained by the substitution of C–H with N in all possible ways, similar as in ref (23). The ST gaps were computed using eqs 10 and 13. Initial tests on systems 1–6 have shown (see Table 2) that CASSCF(14,14) orbitals satisfy conditions required to invert the ST gaps via the sp mechanism to a greater extent than HF orbitals, and unlike the latter, they have led to obtaining negative gaps for all molecules. This suggests that correlated orbitals are a better choice for INVEST screening. Guided by this finding, we have used KS-DFT orbitals from ground state BLYP^42−44^ calculations for tests on our extended set of molecules. In Figure 3, we present ST energy gaps obtained without spin polarization (see eq 4) and with spin polarization fully accounted for (eq 13) compared with the EOM-CCSD values. Evidently, employing only the HOMO–LUMO exchange integral for INVEST prescreening does a poor job as hardly any correlation between Δ*E*_ST_^0^ and EOM-CCSD ST gap values can be seen. Shifting the Δ*E*_ST_^0^ values by −0.30 eV and keeping only molecules with resulting negative gaps would leave us with a too large set: it would include not only molecules with EOM-CCSD-predicted negative gaps but also most of those with positive gaps (see the left panel in Figure 3). Thus, a criterion based on Δ*E*_ST_^0^ values is not sufficiently selective in screening for INVEST systems. A satisfactory correlation is obtained by employing the full sp_π_ model with KS-DFT orbitals and shifting the Δ*E*_ST_^sp_π_^ energies by +0.2 eV. This value is recommended in future INVEST prescreening calculations, as it results in a good overlap between the subsets of molecules with positive and negative gaps obtained from the sp model and EOM-CCSD, as shown in Figure 3, right panel. Notice that the correlation is worse with HF orbitals (see the right panel of Figure 13 in Supporting Information).

**Figure 4:**
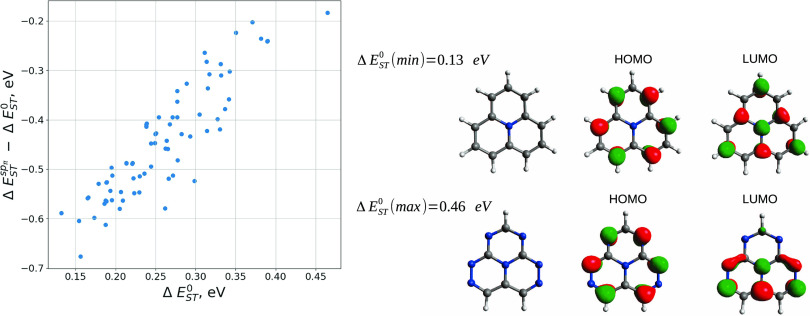
Left panel: Contributions from spin polarization to ST energy gaps (second term on the right-hand side of eq 13) vs ST energy gaps without spin polarization (eq 4). Right panel: Molecules corresponding to the lowest and highest HL exchange interaction. Results obtained with BLYP ground state orbitals.

Another observation following from calculations on the extended set of molecules is that the magnitude of the sp effect decreases linearly with the increasing HL exchange interaction (see Figure 4). Therefore, larger HL integrals correspond to lower spin polarization, both effects being detrimental to obtaining negative ST gaps. Apparently, sp for the considered isoelectronic molecules is as sensitive as HL exchange integrals to the composition and distribution of carbon and nitrogen atoms in heptazine derivatives.

In ref (13), low HL exchange interaction has been associated with high symmetry point groups *D*/*C*_3*h*_, *C*_3*v*_, *C*_2*v*_. In particular, the existence of the σ_*v*_ plane was identified as critical in minimizing the HL overlap. Since, as we have shown, minimization of the latter is accompanied by maximization of the sp effect, in general, it seems to be a good strategy to account for symmetry while designing INVEST molecules. However, symmetry cannot be used as the only criterion in high-throughput screening. Recall that system 4 does not possess σ_*v*_ plane as a symmetry element (Figure 1), but the corresponding ST gap is negative. In contrast, the molecule with the largest HL exchange integral and most positive Δ*E*_*ST*_^sp_π_^ gap (Figure 4) belongs to the *C*_2*v*_ point group.

Results obtained on the extended set show that a simple spin polarization model considered in this section, used with KS-DFT orbitals, can serve as a computationally inexpensive predictor of INVEST molecules. Notice that for systems 1–6 a common-denominator approximation adopted in eq 11 yields energy gaps in good agreement with those obtained if sp from all π orbital pairs is included as in eq 10 (see Table 1 in Supporting Information). Thus, further simplification of the model is achievable if eq 11 is used instead of eq 10. Compared to predictors used in ref (23), based on a double-hybrid DFT functional, the proposed model is computationally more efficient (it requires performing a ground state calculation with a semilocal functional) and above all physically meaningful, as it explicitly includes the sp effect responsible for the gap inversion.

## Effect of Dynamic Correlation Energy

CAS wave functions should effectively capture the sp effect. One expects that expanding the active space from (2,2), two electrons on *H* and *L* orbitals, via (6,6), six electrons on *H*, *L*, and two pairs of degenerate orbitals *H* – 1, *H* – 2 and *L* + 1, *L* + 2, up to (14,14), all π electrons on all π orbitals, would lead to ST energy gaps that reflect the Δ*E*_*ST*_^0^ > Δ*E*_ST_^sp_12_^ > Δ*E*_ST_^sp_π_^ relation observed for PT-based models, namely
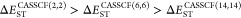
14Indeed, CASSCF energy gaps presented in Table 3 agree with the predicted inequality relations (eq 14) for each system (see the column denoted as CAS). Going from CAS(2,2) to CAS(6,6) lowers the gap by as much as 0.6–1.9 eV. Including all π orbitals as active, in the CASSCF(14,14) model, reduces the gaps by another 0.3–0.5 eV. The Δ*E*_ST_^CASSCF(14,14)^ values are all negative and correspond well with the Δ*E*_ST_^sp_π_^ results obtained using CASSCF(14,14) orbitals (cf. Table 2). We conclude that doubly excited determinants {*iaHL*}, where *i* < *H* and *a* > *L* are π orbitals, are the main contributors to the wave function, after the leading singly excited {*HL*} determinant. In Table 1, we report sums of squares of pertinent CI coefficients for systems 2 and 4. The contribution of the aforementioned double excitations is up to 15%. This is far less than a contribution from {*HL*} determinants amounting to ca. 70%, but it determines the ST gap inversion. It also stresses the importance of having a small value of the HOMO–LUMO exchange integral to achieve gap inversion.

**Figure 5:**
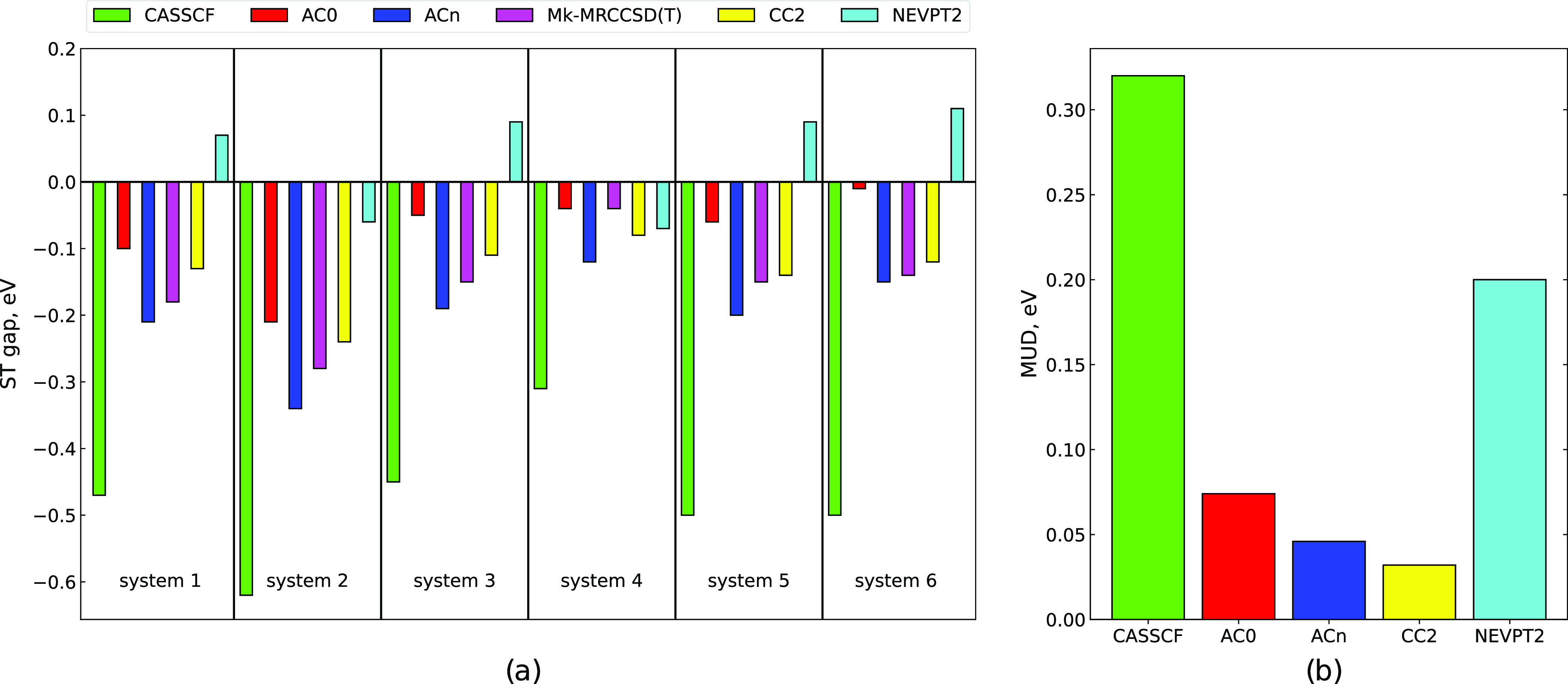
S_1_–T_1_ energy gaps for systems 1–6, panel (a). Mean unsigned deviations of the gaps with respect to Mk-MRCCSD(T) values, panel (b). CASSCF, AC0, ACn, and NEVPT2 results correspond to the (14,14) active space.

A comparison between CASSCF(14,14) and Mk-MRCCSD(T) reference values (cf. Table 3) reveals that CASSCF gaps are too negative with the mean unsigned deviation exceeding 0.3 eV (see also Figure 5). To correct CASSCF for the missing dynamic correlation energy, we use the multireference adiabatic connection (AC) approach,^32,33,45−49^ which recently has been successfully applied to predicting singlet–triplet gaps of biradicals.^34^ Two AC variants are employed in this work: AC0 and ACn. The first one is based on linearizing the adiabatic connection integrand.^32,49^ ACn is free of such an approximation and is expected to yield more accurate predictions.^34^ It is worth mentioning that the computational cost of both AC0 and ACn scales with the fifth power of the system size, AC0 being more efficient than ACn due to a smaller prefactor. AC methods rely only on 1- and 2-electron reduced density matrices (1- and 2-RDMs, respectively), which makes them computationally more efficient in treating large active spaces compared with canonical multireference perturbation theory methods.^48^

AC0 (ACn) ST gaps are computed by adding dynamic correlation correction to the CASSCF gap according to
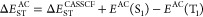
15where *E*^AC^(S_1_) and *E*^AC^(T_1_) are AC0 (ACn) adiabatic connection correlation energies computed from CASSCF 1,2-RDMs for the S_1_ and T_1_ states, respectively. Inspection of the AC0 and ACn energy gaps in Table 3 shows that accounting for dynamic correlation energy counteracts the spin polarization effect. While spin polarization lowers the singlet state energy more than the triplet, leading to a negatively valued ST gap, dynamic correlation shifts the S_1_ energy upward relative to T_1_. In the case of AC0, this effect is overestimated, leading to too small gaps. AC0 gaps computed for CASSCF(6,6) change the sign back to positive for systems 1, 3, 5, and 6. Combining AC0 with CASSCF(14,14) retains the negative sign of the gaps, but their magnitudes are underestimated compared with Mk-MRCCSD(T) values (see also Figure 5). The best accuracy is obtained if ACn is applied together with a CASSCF(14,14) model: the resulting gap values agree up to 0.04 eV with the Mk-MRCCSD(T) reference. AC correlation energy computed for CASSCF(2,2) models yields poor results. This is expected since the CASSCF(2,2) does not contain doubly excited determinants, which AC approximations cannot amend. Ideally, the CASSCF model should include all of the spin polarization, leaving only dynamic correlation energy for adiabatic connection. ACn combined with CASSCF(14,14) proves that this strategy leads to accurate predictions.

In addition to AC, in Table 3, we present the results of NEVPT2 calculations.^35^ Although our previous studies show that NEVPT2 is typically as accurate as AC methods, in particular AC0,^32,45^ for heptazine-based systems the performance of NEVPT2 is inferior. The NEVPT2 energy gap values do not show a systematic improvement when the CAS(*n*,*n*) model is extended. Quite contrary, the behavior is erratic and the gaps are of the wrong sign, except for systems 2 and 4, even if the CAS(14,14) model is employed (cf. Table 3). NEVPT2 gaps deviate on average by 0.2 eV from the reference values (see Figure 5).

The presented results show that two factors are equally important for the accurate prediction of ST gap inversion: accounting for a modest contribution of double excitations and proper treatment of the dynamic correlation. Apparently, single-reference methods such as CC2 or ADC(2)^10,24^ are capable of predicting ST gaps as accurately as multireference approaches. The CC2 energy gaps taken from ref (24) (see Table 3 and Figure 5) stay in good agreement with the multireference CCSD(T) results deviating from the latter by only 0.03 eV. Since the contribution from double excitations in the wave function is substantial, amounting to 15% (see Table 1), single-reference CC methods may owe their good performance in gap prediction to error cancellation. Finally, we notice that the energy gaps predicted by both single- and multireference methods are quite insensitive to the basis set used (compare the results from Table 3 and Table 2 in Supporting Information).

## Summary and Conclusions

**Figure 6:**
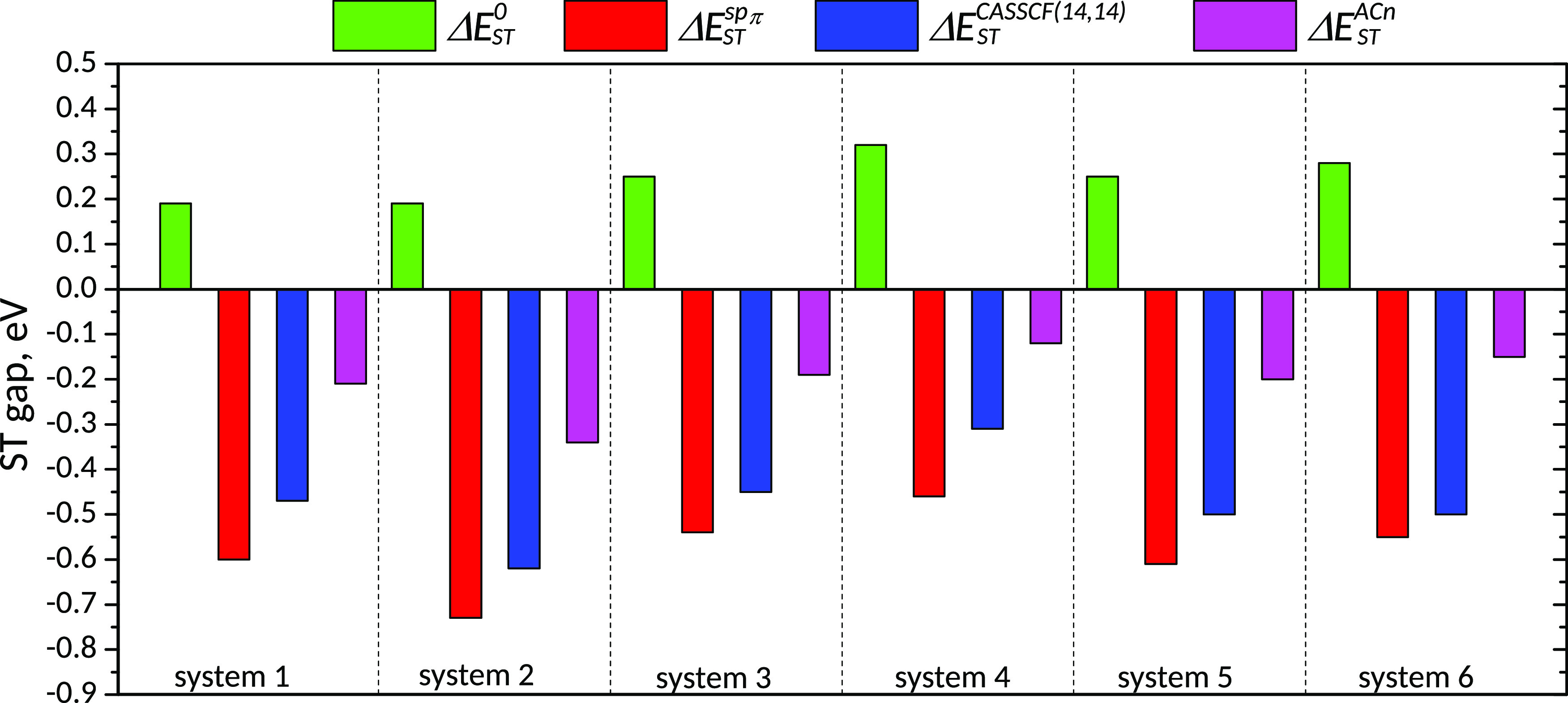
S_1_–T_1_ energy gaps for systems 1–6. 0: no spin polarization (eq 4); sp_π_: PT-based model accounting for spin polarization (eq 13); CASSCF: spin polarization from CASSCF(14,14); ACn: spin polarization at the CAS(14,14) level and dynamic correlation via adiabatic connection (eq 15).

We have investigated the sources of S_1_–T_1_ energy gap inversion in heptazine-based molecules, focusing on the effect of dynamic spin polarization.^22^ We have found that spin polarization, which is equivalent to including doubly excited determinants involving HOMO, LUMO, and any two of the other π orbitals in the wave function expansion, drives the gap inversion. Our findings are summarized in Figure 6. While ignoring spin polarization leads to positive ST gaps (green bars in Figure 6), accounting for this effect alone results in gaps that are too negative (red and blue bars in Figure 6). For a quantitative ST gap prediction, it is essential to consider both spin polarization and accurately treat dynamic correlation energy. Dynamic correlation closes the gap, exerting an effect opposite to that of sp (see pink bars in Figure 6). Comparing several multiconfigurational wave function approaches that account for dynamic correlation, we recommend AC-based methods as having the best accuracy/cost ratio. Among them, the ACn method combined with CASSCF(14,14) was able to reproduce accurate ST gaps for all studied heptazine derivatives.

In the investigated systems, the contribution of double excitations in wave functions varies from 6 to 15%. The effect of double excitations on the relative energies of the S_1_ and T_1_ states can be apparently captured with single-reference coupled-cluster methods. Indeed, we reported a good agreement between CC2 and multireference approaches [ACn and Mk-MRCCSD(T)] in predicting the ST gaps. The agreement may result from error cancellation, as single-reference methods do not fully account for both double-excitation and dynamic correlation effects. As we have shown, including only double excitations in wave functions results in too negative ST gaps. This effect is compensated by accounting for dynamic correlation. Improving a low-order method by increasing the level of the dynamic correlation energy, which is not accompanied by an increased inclusion of double excitations, will erroneously decrease the magnitude of the ST gap, or even change its sign to positive. The latter has been recently reported by Dreuw et al.^50^

We have proposed a simple model for selecting INVEST systems that accounts for two critical factors that favor negative gaps: vanishing HL exchange integral and spin polarization. In this approach, the ST gap is approximated via a PT-based expression involving orbital energies and exchange integrals. When KS-DFT orbitals are employed, the model is capable of efficient and accurate prescreening for INVEST candidates, as we demonstrated on a test set of ∼100 heptazine derivatives. We have found a previously unknown linear correlation between the sp contribution to the ST gap and the magnitude of the HOMO–LUMO (HL) exchange integral. It implies that the quantitative contribution to the gap from the effect of spin polarization is negligible if the HL exchange interaction is relatively large. Our future work will include further validation of the proposed spin-polarization-based model for a broader set of INVEST molecules, going beyond heptazine-based systems. Having the advantage of being low-cost to compute, the model can be used in high-throughput screening for INVEST systems. It can also be used for the prediction of ST gap inversion by machine-learning algorithms.

## Supporting Information Available

Expressions: Hamiltonian matrix elements of doubly excited states, energy differences of doubly excited states. Tables: ST energy gaps from sp models, ST energy gaps in cc-pVDZ and cc-pVQZ basis sets, singlet- and triplet-state energies for systems 1–6, energy characteristics for all heptazine-derived systems from the extended set. Figures: CASSCF(14,14) orbitals for the S_1_ and T_1_ states, ST energy gaps from sp models with HF orbitals vs EOM-CCSD for an extended set of molecules. Geometries of systems 1–6 (PDF)
Geometries_xyz_extended_set (ZIP)
