# Supplementary material for: Role of Spin Polarization and Dynamic Correlation in Singlet–Triplet Gap Inversion of Heptazine Derivatives
Source: J Chem Theory Comput. 2023 Oct 21;19(21):7606–16. doi: 10.1021/acs.jctc.3c00781 (PMC10653106; doi:10.1021/acs.jctc.3c00781)
Supplement: Supplementary file 1 — ct3c00781_si_001.pdf [file ct3c00781_si_001.pdf]

The role of spin polarization and dynamic correlation in singlet-triplet gap inversion of heptazine derivatives – Supporting Information

Daria Drwal,[†,‖] Mikulas Matousek,[‡,¶,‖] Pavlo Golub,[‡] Aleksandra Tucholska,[†] Michał Hapka,[§] Jiri Brabec,[‡] Libor Veis,[*,‡] and Katarzyna Pernal[*,†]

†*Institute of Physics, Lodz University of Technology, ul. Wolczanska 219, 90-924 Lodz, Poland*

‡*J. Heyrovský Institute of Physical Chemistry, Academy of Sciences of the Czech Republic, v.v.i., Dolejškova 3, 18223 Prague 8, Czech Republic*

¶*Faculty of Mathematics and Physics, Charles University, Prague, Czech Republic*

§*Faculty of Chemistry, University of Warsaw, ul. L. Pasteura 1, 02-093 Warsaw, Poland*

‖*Contributed equally.*

E-mail: libor.veis@jh-inst.cas.cz; pernalk@gmail.com

Expressions

Spin-free Hamiltonian matrix elements of doubly excited states

$$
\begin{aligned}
\left\langle \Psi_S^0 | \hat{H} | \Psi_S^1 \right\rangle &= -\sqrt{\frac{3}{2}} \left(\langle aH|Hi \rangle - \langle aL|Li \rangle \right) \\
\left\langle \Psi_S^0 | \hat{H} | \Psi_S^{1'} \right\rangle & \frac{1}{\sqrt{2}} \left(\langle aH|Hi \rangle + \langle aL|Li \rangle \right) \\
\left\langle \Psi_T^0 | \hat{H} | \Psi_T^1 \right\rangle &= \frac{1}{\sqrt{2}} \left(\langle aH|Hi \rangle - \langle aL|Li \rangle \right) \\
\left\langle \Psi_T^0 | \hat{H} | \Psi_T^2 \right\rangle &= -\left(\langle aH|Hi \rangle + \langle aL|Li \rangle \right) \\
\left\langle \Psi_T^0 | \hat{H} | \Psi_T^{1'} \right\rangle &= \frac{1}{\sqrt{2}} \left(\langle aH|Hi \rangle + \langle aL|Li \rangle \right)
\end{aligned}
$$

Energy differences of doubly excited states

We define the Coulombic part as

$$
\begin{aligned}
\Delta E_{\text{Coul}} &= \epsilon_a - \epsilon_i - \langle ai|ai \rangle - \langle Ha|Ha \rangle - \langle iL|iL \rangle + \langle iH|iH \rangle + \langle aL|aL \rangle \\
\epsilon_a &= \langle a|h|a \rangle + \sum_{j \leq H} \left(2 \langle aj|aj \rangle - \langle aj|ja \rangle \right) \\
\epsilon_i &= \langle i|h|i \rangle + \sum_{j \leq H} \left(2 \langle ij|ij \rangle - \langle ij|ji \rangle \right)
\end{aligned}
$$

which greatly simplifies the final (spin-free) expressions for differences of state-energies

$$
\begin{aligned}
E_S^0 - E_T^0 &= 2 \langle HL|LH \rangle \\
E_S^1 - E_S^0 &= \Delta E_{\text{Coul}} + \frac{3}{2} \langle iL|Li \rangle + \frac{3}{2} \langle Ha|aH \rangle - \frac{1}{2} \langle iH|Hi \rangle - \frac{1}{2} \langle La|aL \rangle - 2 \langle HL|LH \rangle \\
E_S^{1'} - E_S^0 &= \Delta E_{\text{Coul}} + \frac{1}{2} \left(\langle iL|Li \rangle + \langle Ha|aH \rangle - \langle iH|Hi \rangle - \langle La|aL \rangle \right) + 2 \langle ai|ia \rangle \\
E_T^1 - E_T^0 &= \Delta E_{\text{Coul}} + \frac{1}{2} \left(\langle iL|Li \rangle + \langle Ha|aH \rangle - \langle iH|Hi \rangle - \langle La|aL \rangle \right) + 2 \langle HL|LH \rangle \\
E_T^2 - E_T^0 &= \Delta E_{\text{Coul}} + \langle iL|Li \rangle + \langle Ha|aH \rangle \\
E_T^{1'} - E_T^0 &= \Delta E_{\text{Coul}} + \frac{1}{2} \left(\langle iL|Li \rangle + \langle Ha|aH \rangle - \langle iH|Hi \rangle - \langle La|aL \rangle \right) + 2 \langle ai|ia \rangle
\end{aligned}
$$

Tables

Table 1: S_1-T_1 energy gaps obtained with spin polarization computed from two pairs of π orbitals, Eq. (12) in the main text, and from all occupied-virtual pairs of π orbitals, Eq. (13) in the main text. Asterisk at HF and CASSCF(14,14) indicates that a common denominator approximation, Eq.(11) in the main text, has been employed. All values in eV.

Orbitals	ST gap	1	2	3	4	5	6
HF*	$\Delta E_{ST}^{sp_\pi}$	-0.43	-0.54	-0.12	0.11	0.09	0.00
	$\Delta E_{ST}^{sp_{12}}$	-0.22	-0.28	0.07	0.29	0.32	0.21
CASCI(14,14)	$\Delta E_{ST}^{sp_\pi}$	-0.43	-0.53	-0.35	0.27	-0.38	-0.32
CASSCF(14,14)*	$\Delta E_{ST}^{sp_\pi}$	-0.72	-0.90	-0.66	-0.56	-0.74	-0.67
	$\Delta E_{ST}^{sp_{12}}$	-0.45	-0.58	-0.40	-0.29	-0.45	-0.39
BLYP	$\Delta E_{ST}^{sp_{12}}$	-0.18	-0.18	-0.06	0.00	-0.01	0.01
	$\Delta E_{ST}^{sp_\pi}$	-0.44	-0.51	-0.33	-0.27	-0.36	-0.29

Table 2: S_1-T_1 gaps in cc-pVDZ and cc-pVQZ basis sets. Multireference calculations employed CASSCF(14,14), except for NEVPT2 for system 4 for which CASSCF(14,13) was used. All values in eV.

System	CAS	AC0	ACn	NEVPT2	CC2
			cc-pVDZ		
1	-0.48	-0.12	-0.23	0.02	-0.15
2	-0.63	-0.23	-0.36	-0.09	-0.26
3	-0.47	-0.09	-0.21	0.04	-0.13
4	-0.33	-0.08	-0.14	-0.09	-0.10
5	-0.51	-0.11	-0.23	0.04	-0.16
6	-0.51	-0.11	-0.23	0.05	-0.15
			cc-pVQZ		
2	-0.62	-0.21	-0.34	-	-

Table 3: Singlet and triplet state energies. All values in Ha.

System	State	CAS	AC0	ACn	NEVPT2	Mk-MRCCSD
CAS(2,2)						
1	S1	-514.18531	-516.50650	-516.32429	-516.26237	-516.66170
	T1	-514.25003	-516.53204	-516.32443	-516.26846	-516.65521
2	S1	-610.16836	-612.71904	-612.45863	-612.49221	-612.84185
	T1	-610.17978	-612.68982	-612.44843	-612.45406	-612.83154
3	S1	-546.18004	-548.57564	-548.36824	-548.35095	-548.46751
	T1	-546.19625	-548.56530	-548.36907	-548.32917	-548.46219
4	S1	-562.17560	-564.60998	-564.40082	-564.39043	-564.75333
	T1	-562.20474	-564.59363	-564.39289	-564.35649	-564.75191
5	S1	-578.18423	-580.64588	-580.41737	-580.41799	-580.78857
	T1	-578.21005	-580.62227	-580.41522	-580.38358	-580.78322
6	S1	-578.17529	-580.64793	-580.41430	-580.42206	-580.78166
	T1	-578.20165	-580.62338	-580.41213	-580.38610	-580.77645
CAS(6,6)						
1	S1	-514.24665	-516.48663	-516.33787	-516.26237	
	T1	-514.23993	-516.49263	-516.33810	-516.25957	
2	S1	-610.24831	-612.68836	-612.47734	-612.45738	
	T1	-610.23705	-612.68773	-612.47217	-612.49458	
3	S1	-546.24466	-548.55440	-548.38353	-548.33081	
	T1	-546.23847	-548.55722	-548.38324	-548.32947	
4	S1	-562.24165	-564.59091	-564.40388	-564.36918	
	T1	-562.24811	-564.57925	-564.40348	-564.34733	
5	S1	-578.25087	-580.63449	-580.43897	-580.40715	
	T1	-578.24637	-580.63588	-580.42702	-580.39814	

System	State	CAS	AC0	ACn	NEVPT2	Mk-MRCCSD
6	S1	-578.24954	-580.62272	-580.43324	-580.39401	
	T1	-578.24131	-580.62560	-580.43042	-580.39573	
			CAS(14,14)			
1	S1	-514.36853	-516.48388	-516.37625	-516.26241	
	T1	-514.35117	-516.48036	-516.36853	-516.26481	
2	S1	-610.39277	-612.68043	-612.52654	-612.45840	
	T1	-610.36982	-612.67275	-612.51412	-612.45622	
3	S1	-546.37567	-548.55025	-548.42682	-548.32404	
	T1	-546.35897	-548.54839	-548.42001	-548.32740	
4	S1	-562.38349	-564.58471	-564.45384	-564.36614	
	T1	-562.37197	-564.58314	-564.44961	-564.36340	
5	S1	-578.39023	-580.62282	-580.48349	-580.40090	
	T1	-578.37170	-580.62068	-580.47629	-580.40408	
6	S1	-578.38483	-580.61388	-580.47530	-580.39263	
	T1	-578.36628	-580.61339	-580.46972	-580.39663	

Orbitals

Orbitals from state specific CASSCF(14,14) calculations for S_1 and T_1 states (for system 4 orbitals for S_1 were obtained from SA-CASSCF(14,14) calculation with two states). Six natural orbitals of highest occupancies are shown for each system except for system 2 for which 14 orbitals are presented for each state.

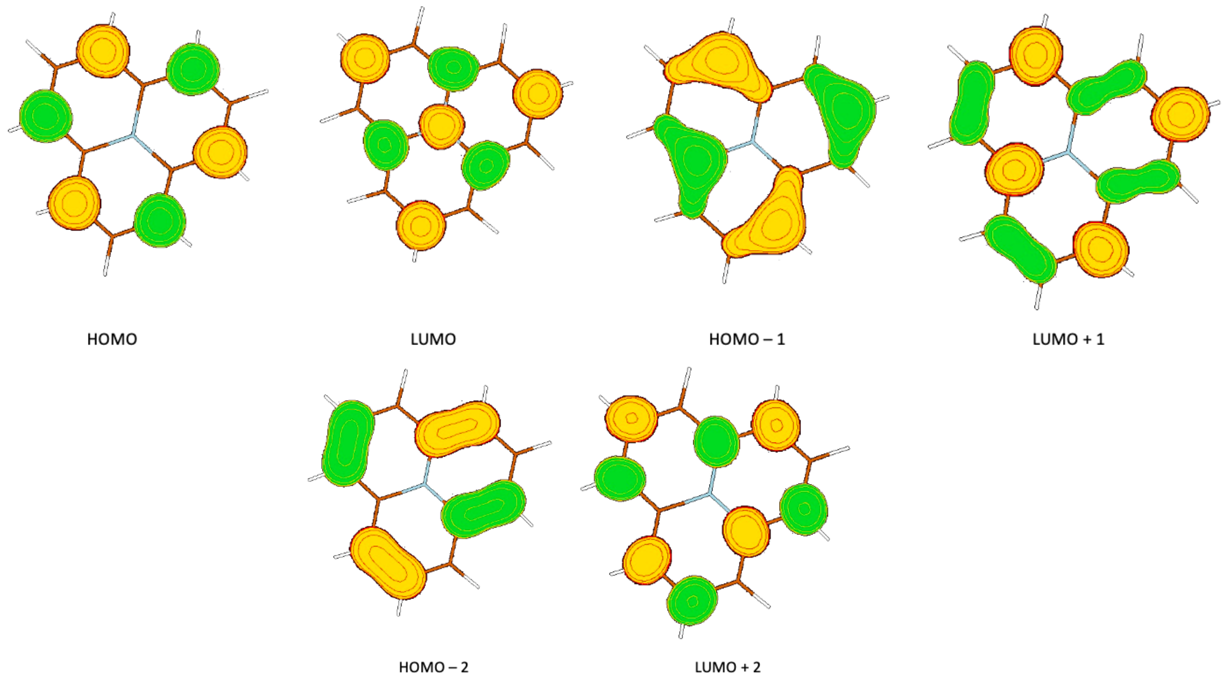

Figure 1: System 1. Singlet state.

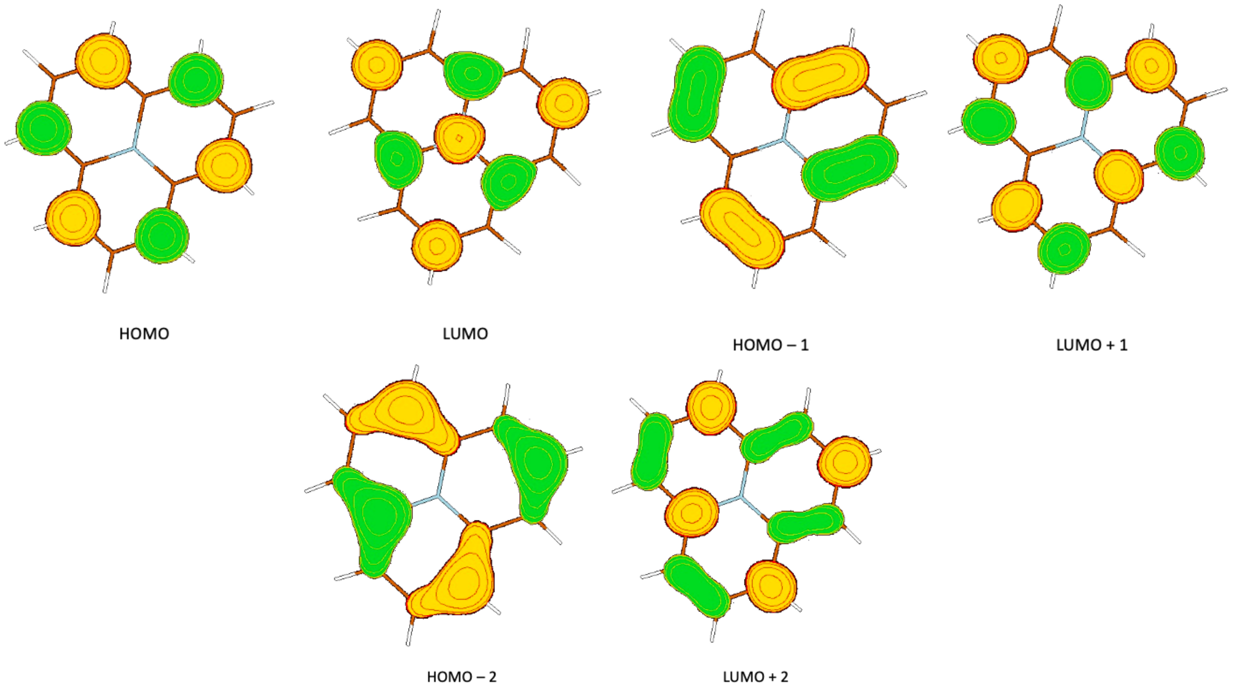

Figure 2: System 1. Triplet state.

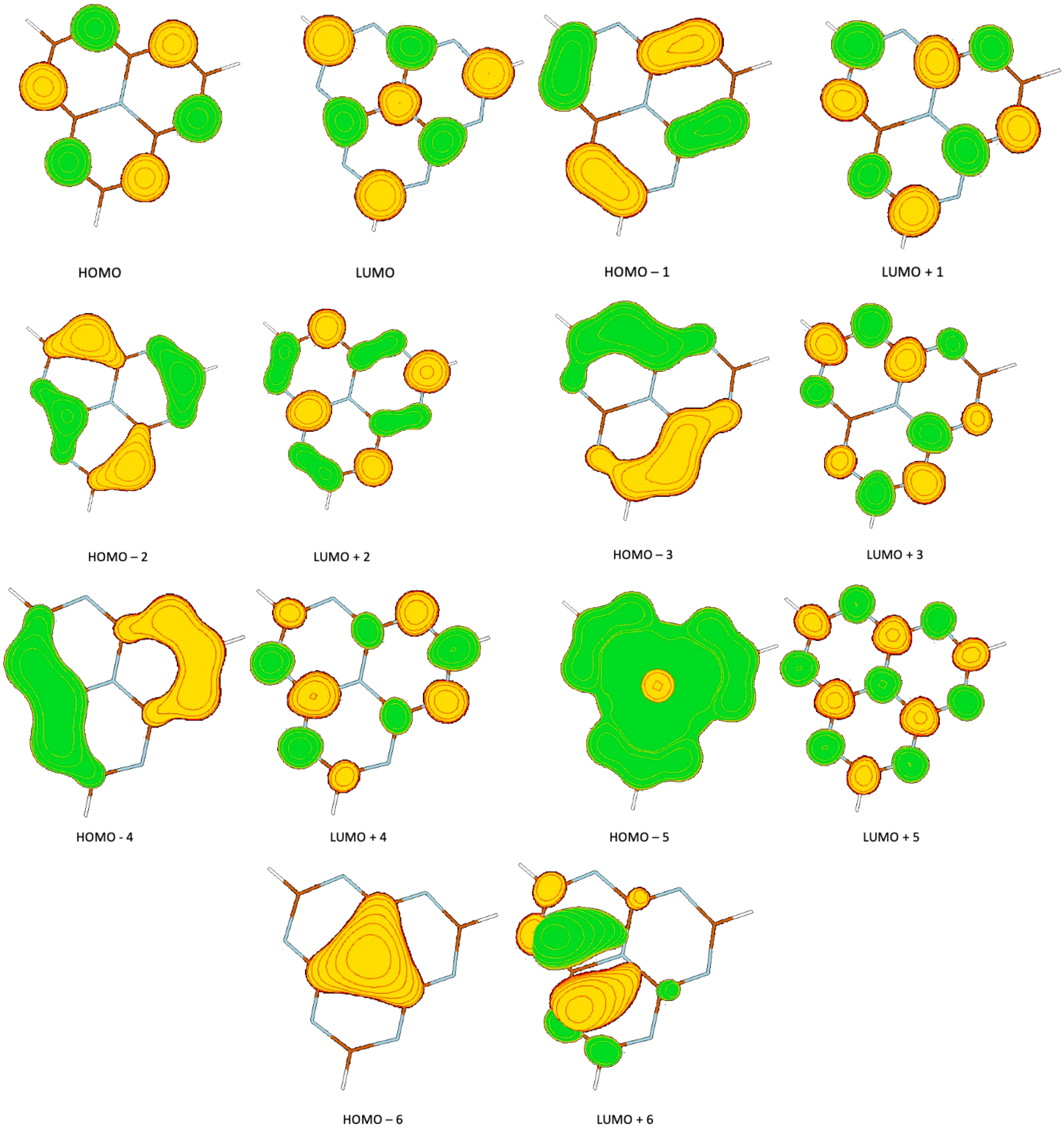

Figure 3: System 2. Singlet state.

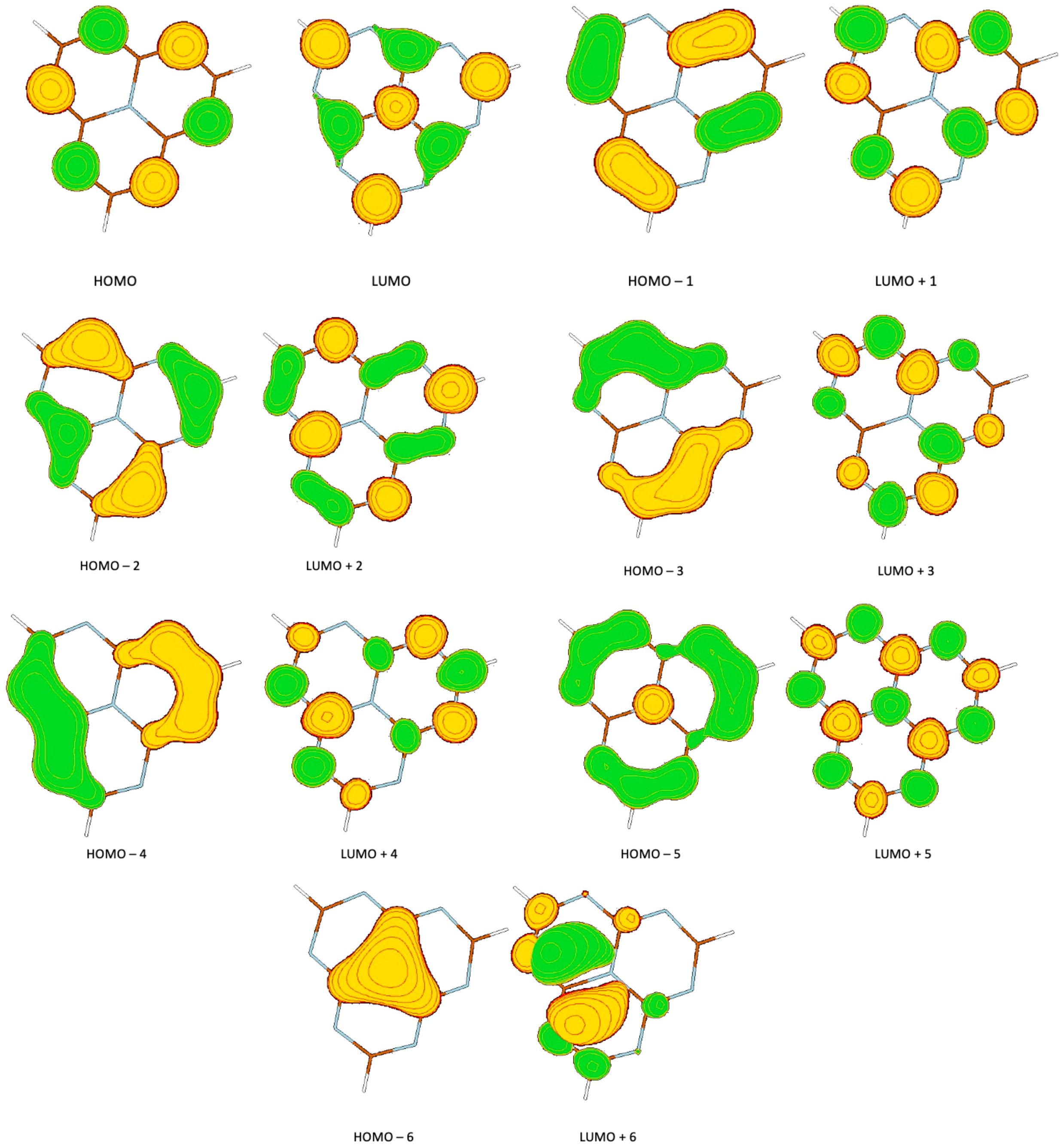

Figure 4: System 2. Triplet state.

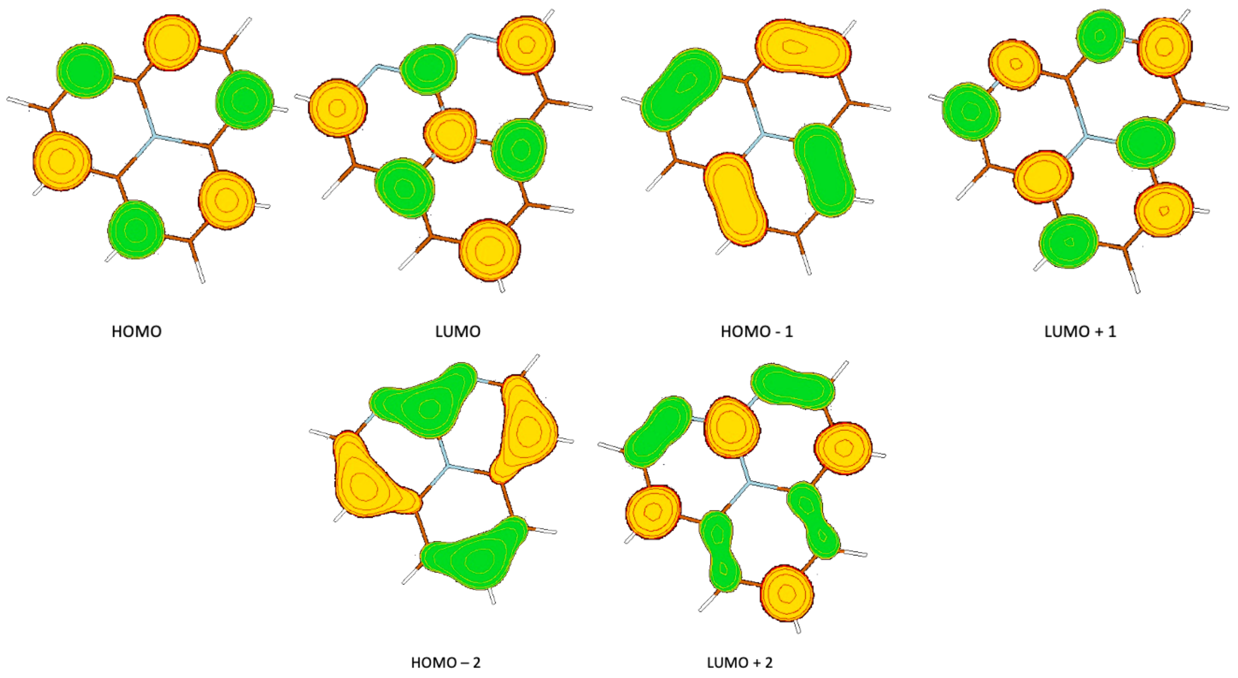

Figure 5: System 3. Singlet state.

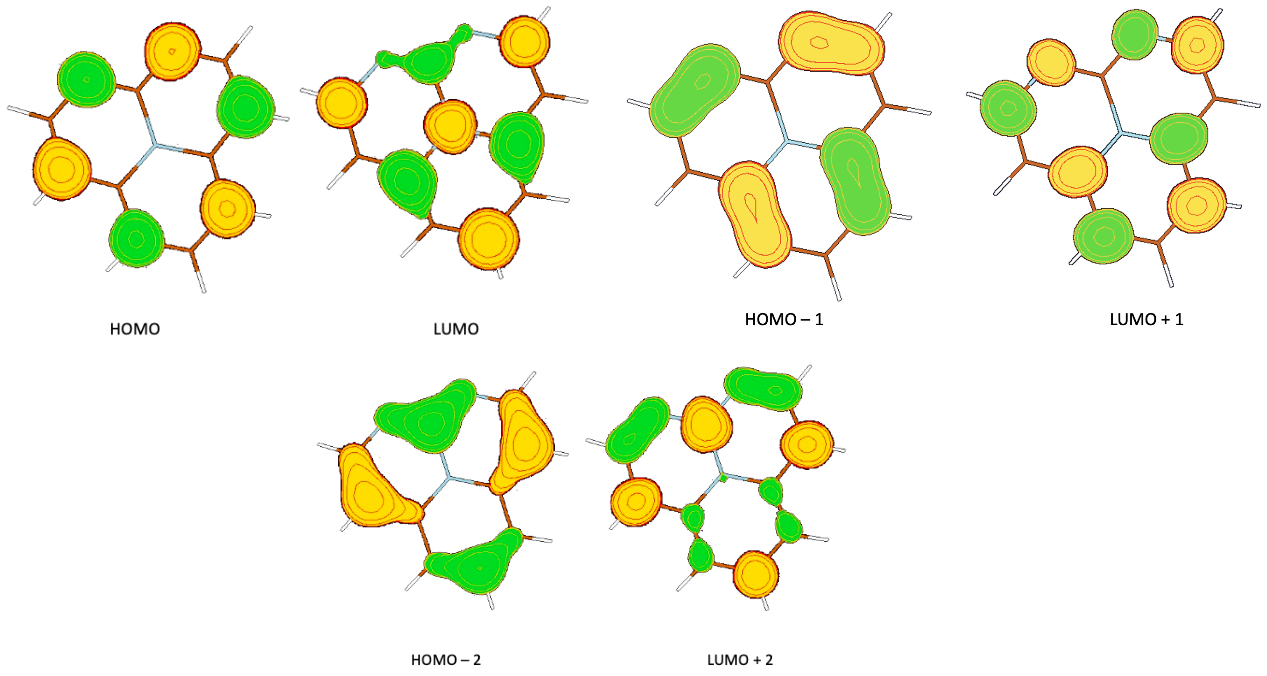

Figure 6: System 3. Triplet state.

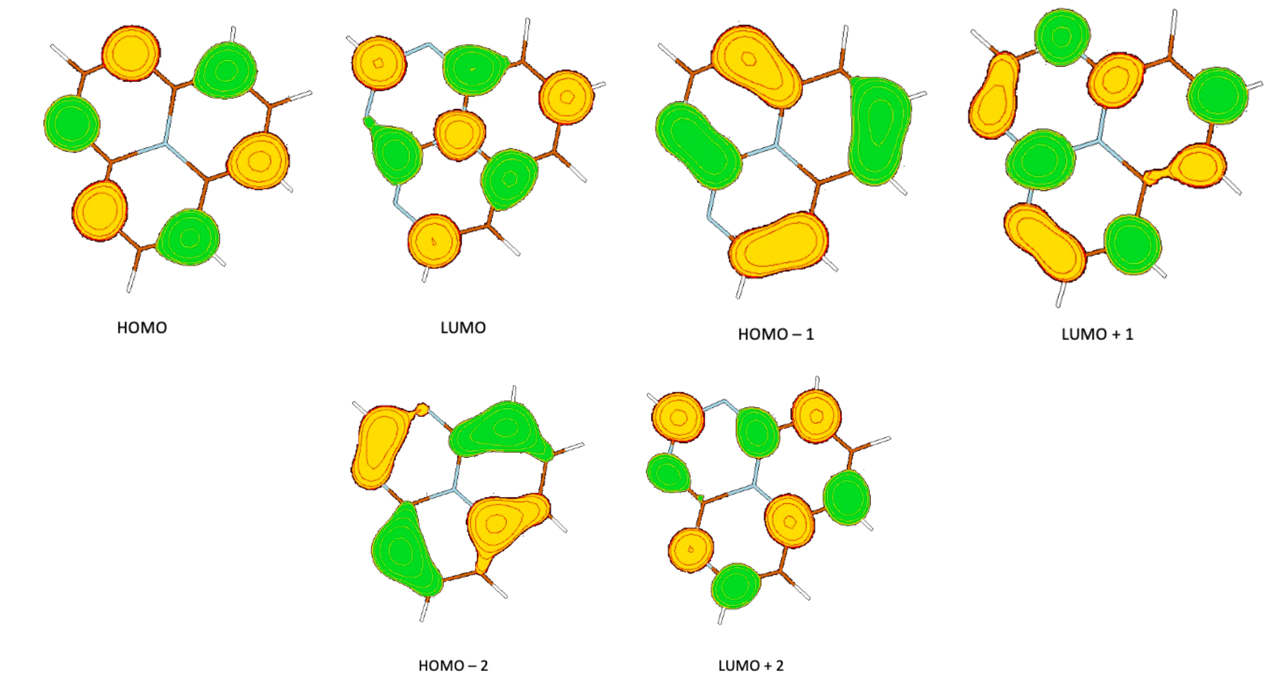

Figure 7: System 4. Singlet state.

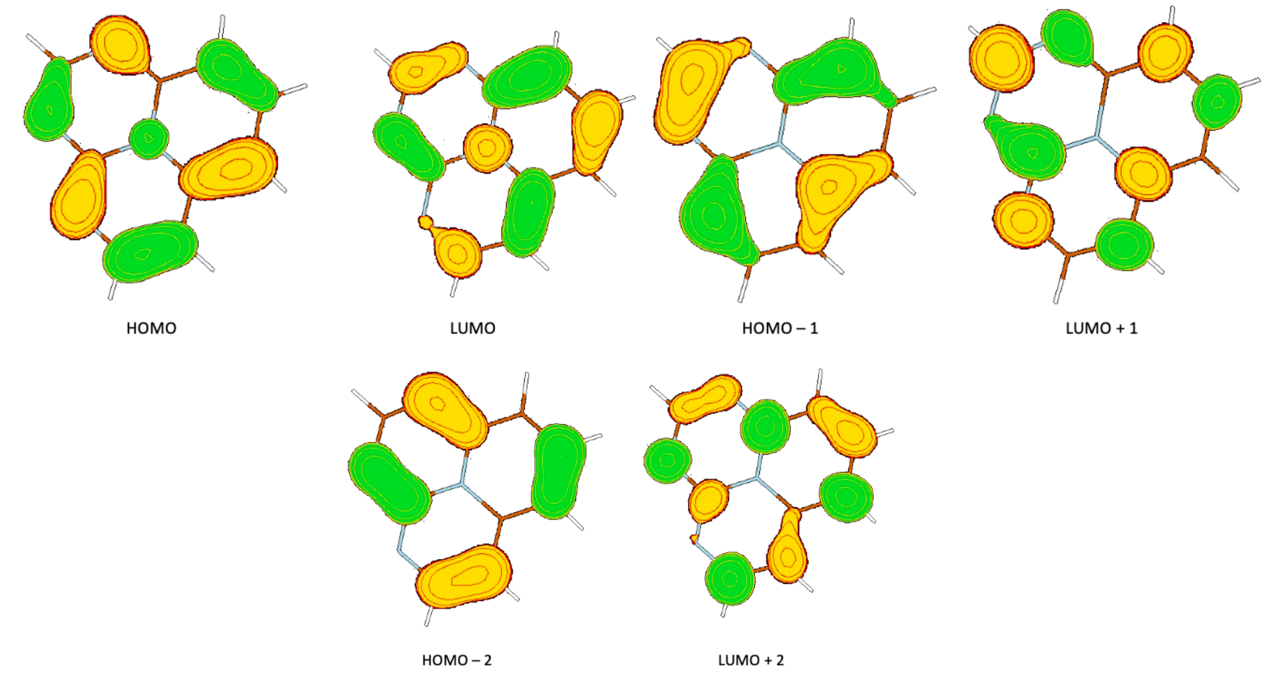

Figure 8: System 4. Triplet state.

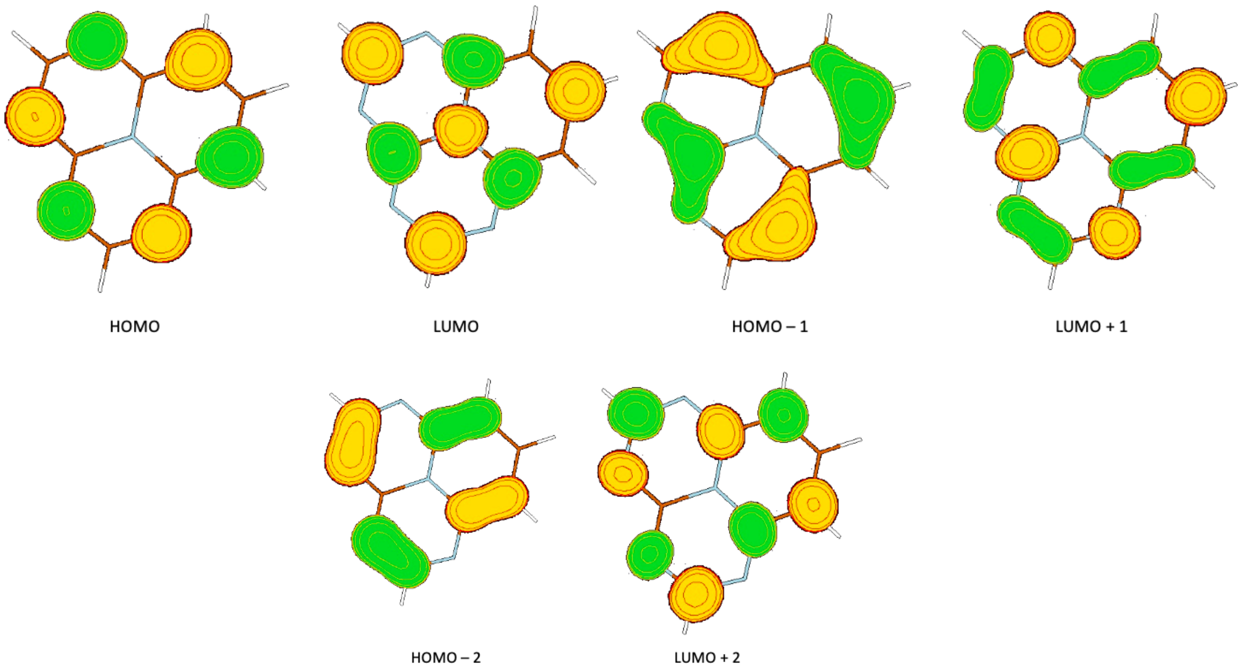

Figure 9: System 5. Singlet state.

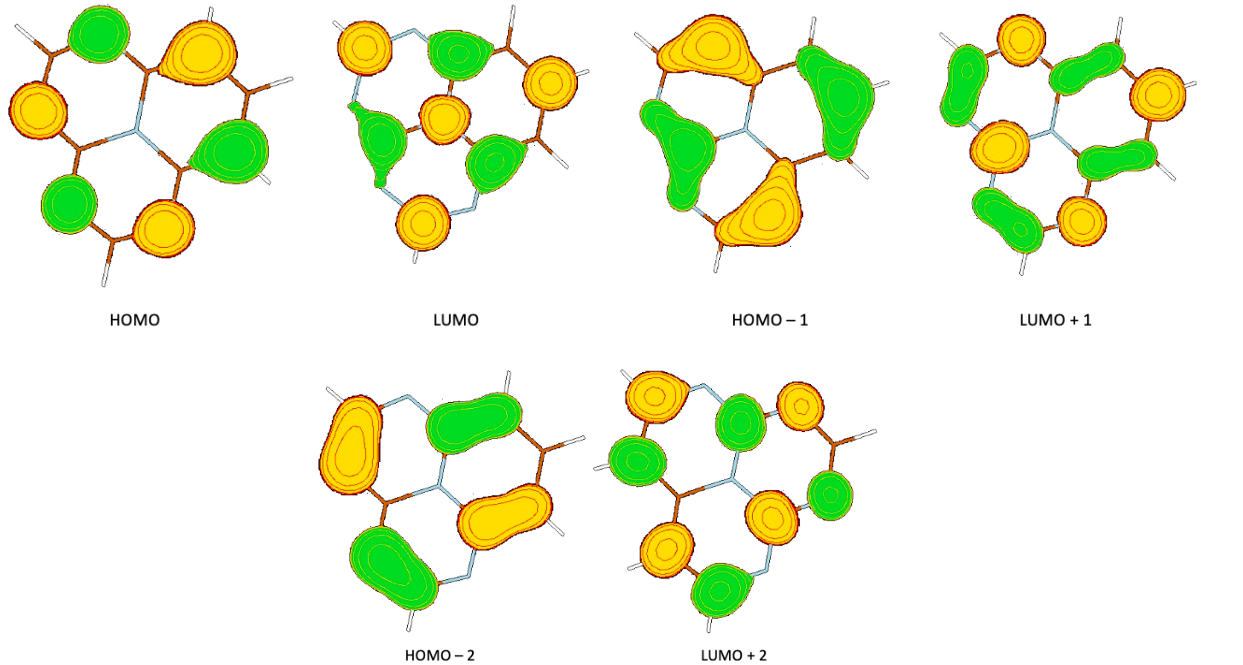

Figure 10: System 5. Triplet state.

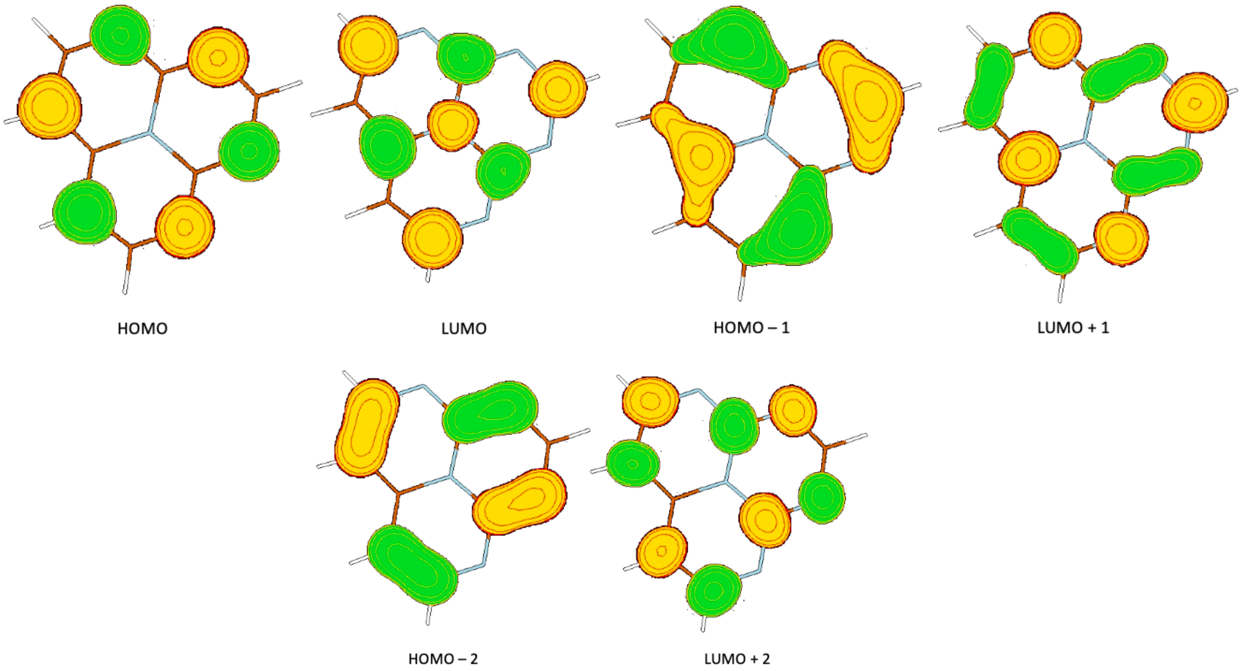

Figure 11: System 6. Singlet state.

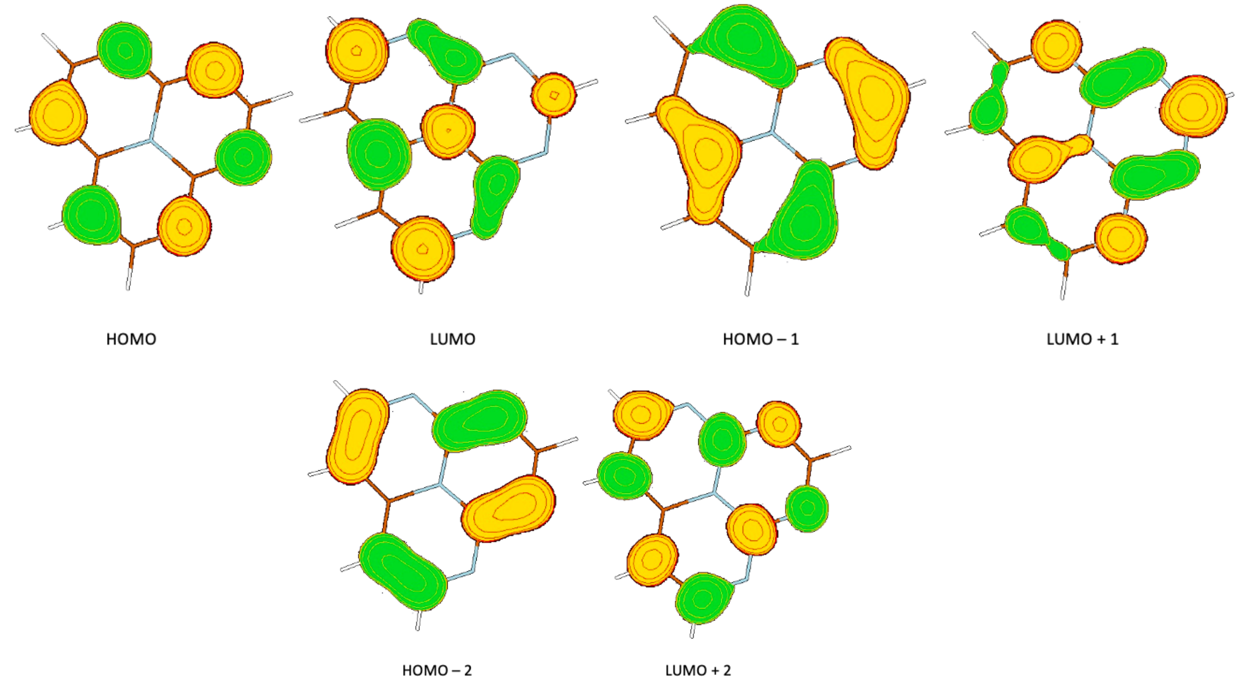

Figure 12: System 6. Triplet state.

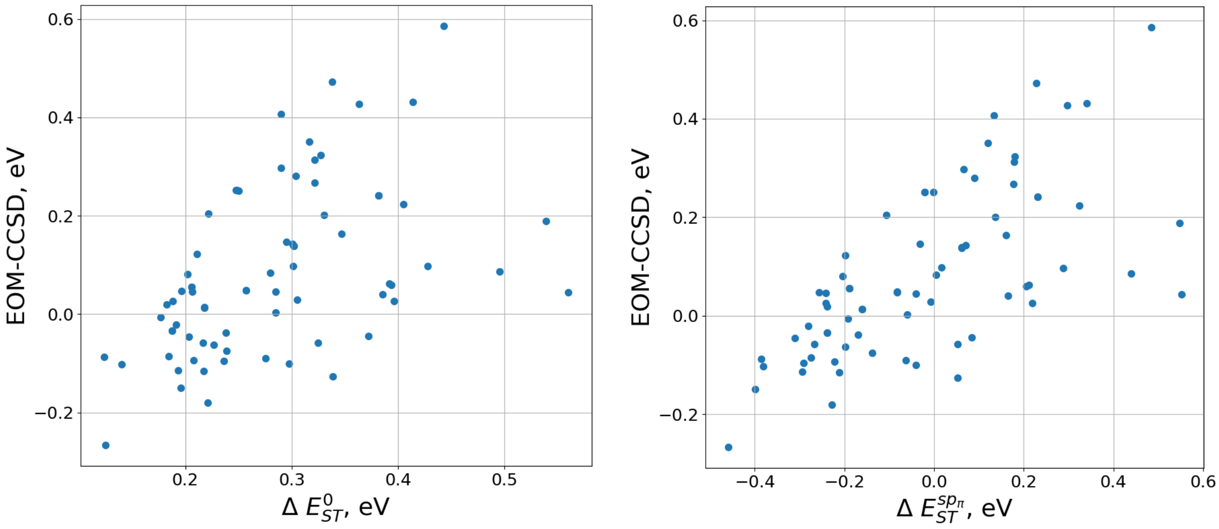

Figure 13: Left panel: ST energy gaps without spin polarization, Eq. (4) in the main text. Right panel: ST energy gaps with spin polarization from all occupied-virtual π orbital pairs, Eqs. (13) and (10) in the main text. Results obtained with restricted HF ground state orbitals vs. EOM-CCSD values for extended test set of molecules.

Geometries of the systems 1-6

Geometries of the molecules 1-6 shown in Figure 1 in the main text.

System 1

N 0.0000000000 -0.0075103534 0.0000000000

C 0.0000000000 1.3973851920 0.0000000000

C 0.0000000000 -2.8107150117 0.0000000000

H 0.0000000000 -3.8954411971 0.0000000000

H -3.3670872718 1.9364599919 0.0000000000

H 3.3670872718 1.9364599919 0.0000000000

H -3.3413554944 -0.5597597650 0.0000000000

H 3.3413554944 -0.5597597650 0.0000000000

H -2.1489809607 -2.6250505700 0.0000000000

H 2.1489809607 -2.6250505700 0.0000000000

C -2.4276691375 1.3941344098 0.0000000000

C 2.4276691375 1.3941344098 0.0000000000

C -2.4236105503 0.0136281711 0.0000000000

C 2.4236105503 0.0136281711 0.0000000000

C -1.1935498443 -2.1169275150 0.0000000000

C 1.1935498443 -2.1169275150 0.0000000000

C 1.2165387210 -0.7098602818 0.0000000000

C -1.2165387210 -0.7098602818 0.0000000000

C 1.2300736123 2.0808930024 0.0000000000

C -1.2300736123 2.0808930024 0.0000000000

H 1.1923888713 3.1623902417 0.0000000000

H -1.1923888713 3.1623902417 0.0000000000

System 2

N 0.0000000000 0.0093346731 0.0000000000

C 0.0000000000 1.4070075907 0.0000000000

C 0.0000000000 -2.5985095237 0.0000000000

H 0.0000000000 -3.6843310325 0.0000000000

N -1.1600248342 2.0623688475 0.0000000000

C -1.2106362019 -0.6896624105 0.0000000000

N -1.1980321921 -2.0218840429 0.0000000000

N 1.1600248342 2.0623688475 0.0000000000

C 1.2106362019 -0.6896624105 0.0000000000

N 1.1980321921 -2.0218840429 0.0000000000

C 2.2584217418 1.3132705846 0.0000000000

N 2.3580561028 -0.0126389450 0.0000000000

C -2.2584217418 1.3132705846 0.0000000000

N -2.3580561028 -0.0126389450 0.0000000000

H -3.1987782274 1.8561551125 0.0000000000

H 3.1987782274 1.8561551125 0.0000000000

System 3

C -1.7154104794 2.2998597786 0.0000000000

N -2.3908340959 1.1463481382 0.0000000000

C -0.3477387781 2.3924053092 0.0000000000

C 0.4149805050 1.2095445483 0.0000000000

N -0.2775609122 0.0000000082 0.0000000000

C -1.7087316122 0.0000000285 0.0000000000

C 0.4149804706 -1.2095445517 0.0000000000

C 1.8159429208 -1.1949818216 0.0000000000

C 2.5105297878 -0.0000000315 0.0000000000

C 1.8159429548 1.1949817784 0.0000000000

H 3.5947518878 -0.0000000470 0.0000000000

H 2.3243229684 2.1503351711 0.0000000000

H 0.1654783854 3.3442146019 0.0000000000

H 2.3243229072 -2.1503352289 0.0000000000

C -0.3477388462 -2.3924052908 0.0000000000

C -1.7154105449 -2.2998597214 0.0000000000

N -2.3908341285 -1.1463480618 0.0000000000

H 0.1654782902 -3.3442145981 0.0000000000

H -2.3262359577 -3.1985847127 0.000000000

H -2.3262358666 3.1985847873 0.0000000000

System 4

C -2.4314987000 1.6324059000 -0.0000000000

N -1.3292401000 2.3606919000 0.0000000000

N -2.5467772000 0.3074545000 -0.0000000000

C -1.3913540000 -0.3767382000 -0.0000000000

N -0.1602693000 0.2819983000 0.0000000000

C -0.1505621000 1.7045290000 0.0000000000

C 1.0381219000 -0.4226079000 0.0000000000

C 1.0057171000 -1.8154535000 -0.0000000000

C -0.2068079000 -2.4882850000 -0.0000000000

C -1.3903747000 -1.7783900000 -0.0000000000

H -0.2265006000 -3.5723273000 -0.0000000000

H -2.3603662000 -2.2564555000 -0.0000000000

H 1.9507714000 -2.3423543000 -0.0000000000

C 2.2288634000 0.3436217000 0.0000000000

C 2.1423994000 1.7037226000 0.0000000000

N 0.9749063000 2.3856237000 0.0000000000

H 3.1797666000 -0.1719795000 0.0000000000

H 3.0387205000 2.3160328000 0.0000000000

H -3.3655160000 2.1885108000 -0.0000000000

System 5

C 0.0000000000 1.3809898743 0.0000000000

N 0.0000000000 -0.0329199737 0.0000000000

C 0.0000000000 -2.7855633651 0.0000000000

H 0.0000000000 -3.8696436170 0.0000000000

N -1.1567984507 2.0363927364 0.0000000000

C -1.2186950450 -0.7046345529 0.0000000000

C -1.2019439853 -2.0981489061 0.0000000000

N 1.1567984507 2.0363927364 0.0000000000

C 1.2186950450 -0.7046345529 0.0000000000

C 1.2019439853 -2.0981489061 0.0000000000

C -2.2720407513 1.2980691945 0.0000000000

N -2.3828851199 -0.0148620542 0.0000000000

H -2.1623249144 -2.5950928799 0.0000000000

C 2.2720407513 1.2980691945 0.0000000000

N 2.3828851199 -0.0148620542 0.0000000000

H 2.1623249144 -2.5950928799 0.0000000000

H -3.2032871920 1.8574810030 0.0000000000

H 3.2032871920 1.8574810030 0.0000000000

System 6

N 0.0000000000 0.0571380583 0.0000000000

C 0.0000000000 1.4421644198 0.0000000000

C 0.0000000000 -2.5691253110 0.0000000000

H 0.0000000000 -3.6555558734 0.0000000000

N 2.3864469928 -0.0221603029 0.0000000000

N -2.3864469928 -0.0221603029 0.0000000000

C 2.3832445311 1.3156920036 0.0000000000

C -2.3832445311 1.3156920036 0.0000000000

C -1.2447303345 2.0856673398 0.0000000000

C 1.2447303345 2.0856673398 0.0000000000

H 1.2798263021 3.1665362708 0.0000000000

H -1.2798263021 3.1665362708 0.0000000000

H 3.3626362651 1.7852611520 0.0000000000

H -3.3626362651 1.7852611520 0.0000000000

C 1.2193441862 -0.6563458824 0.0000000000

C -1.2193441862 -0.6563458824 0.0000000000

N 1.1952853736 -1.9947782277 0.0000000000

N -1.1952853736 -1.9947782277 0.0000000000

Extended test set of heptazine-derived molecules

Data used in Figures 3 and 4 in the main text.

Table 4: Energy characteristics for all heptazine-derived systems. ΔE_{S_1} – excitation energy for S_1 state, ΔE_{T_1} – excitation energy for T_1 state, ΔE_{ST}^{EOM} – EOM-CCSD S_1–T_1 gaps, ΔE_{ST}^{0} – S_1–T_1 gaps obtained without spin polarization, $\Delta E_{ST}^{sp_\pi}$ – S_1–T_1 gaps obtained with spin polarization computed from two pairs of π orbitals. All energies are in eV.

System	ΔE_{S_1}	ΔE_{T_1}	ΔE_{ST}^{EOM}	ΔE_{ST}^{0}	$\Delta E_{ST}^{sp_\pi}$
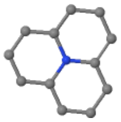	1.0893	1.1766	-0.0872	0.1320	-0.4568
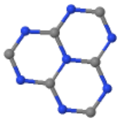	2.7909	3.0574	-0.2664	0.1562	-0.5204
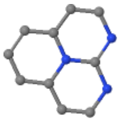	1.6558	1.7308	-0.0750	0.1934	-0.3500
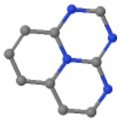	2.0127	2.0711	-0.0583	0.2220	-0.2962
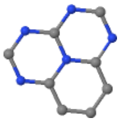	2.2515	2.3780	-0.1266	0.2050	-0.3747
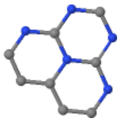	2.2090	2.3097	-0.1007	0.2230	-0.3256
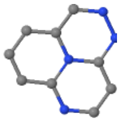	2.1248	1.8118	0.3129	0.2887	-0.0384
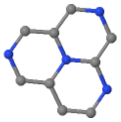	1.4331	1.2323	0.2008	0.2711	-0.1237
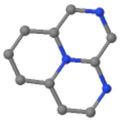	1.3915	1.3434	0.0481	0.2134	-0.2746

Continued on next page

Table 4 – continued from previous page

System	ΔE_{S_1}	ΔE_{T_1}	ΔE_{ST}^{EOM}	ΔE_{ST}^{0}	$\Delta E_{ST}^{sp_\pi}$
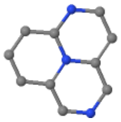	1.4671	1.3282	0.1388	0.2504	-0.1771
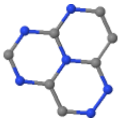	2.3586	2.2158	0.1428	0.3151	-0.0212
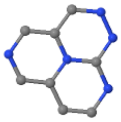	1.5901	1.3665	0.2236	0.3320	0.0214
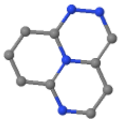	1.5217	1.4409	0.0808	0.2491	-0.1978
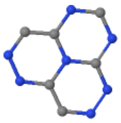	2.6231	2.3559	0.2672	0.3314	0.0438
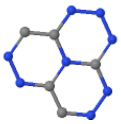	2.7421	2.3914	0.3506	0.3427	0.0408
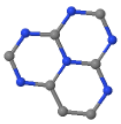	2.5265	2.7073	-0.1808	0.1875	-0.4249
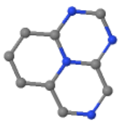	1.5883	1.5624	0.0259	0.2652	-0.1936
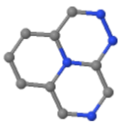	1.7298	1.4323	0.2975	0.2539	-0.1406
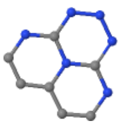	1.9912	2.1058	-0.1145	0.2091	-0.3545
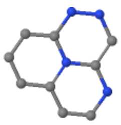	2.0209	1.8161	0.2048	0.2383	-0.1757

Continued on next page

Table 4 – continued from previous page

System	ΔE_{S_1}	ΔE_{T_1}	ΔE_{ST}^{EOM}	ΔE_{ST}^{0}	$\Delta E_{ST}^{sp_\pi}$
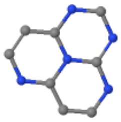	2.3434	2.4372	0.0938	0.1952	-0.3674
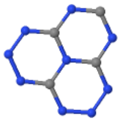	2.2878	2.3833	-0.0955	0.2984	-0.2256
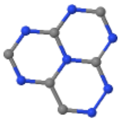	2.4793	2.4197	0.0596	0.3271	-0.0720
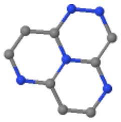	2.4331	2.1818	0.2513	0.2770	-0.0645
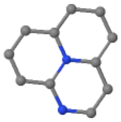	1.4853	1.5198	-0.0345	0.1648	-0.3940
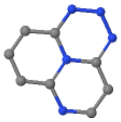	1.8309	1.7855	0.0454	0.2440	0.2507
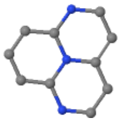	1.6394	1.7421	-0.1027	0.1544	-0.4499
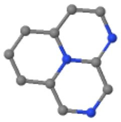	1.3907	1.3431	0.0475	0.2135	-0.2741
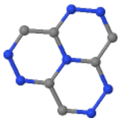	3.0104	2.5388	0.4716	0.3503	0.1263
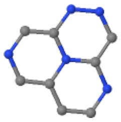	2.0721	1.7919	0.2802	0.2769	-0.0865
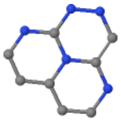	1.9981	1.9429	0.0552	0.2206	-0.2682

Continued on next page

Table 4 – continued from previous page

System	ΔE_{S_1}	ΔE_{T_1}	ΔE_{ST}^{EOM}	ΔE_{ST}^{0}	$\Delta E_{ST}^{sp_\pi}$
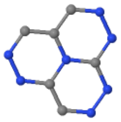	2.0396	1.6081	0.4315	0.3814	0.1453
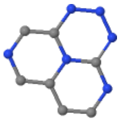	1.6750	1.6347	0.0403	0.2929	-0.1410
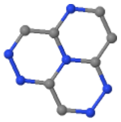	2.6967	2.2897	0.4070	0.3114	0.0473
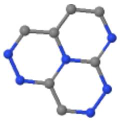	2.2258	1.9022	0.3236	0.3174	0.0098
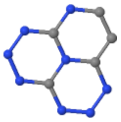	2.1188	2.1648	-0.0461	0.2717	-0.2412
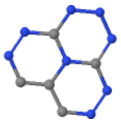	1.6627	1.5659	0.0968	0.3366	-0.0418
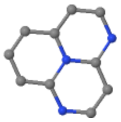	1.8864	1.8733	0.0131	0.1887	-0.3382
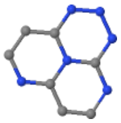	2.1822	2.1348	0.0474	0.2622	-0.1958
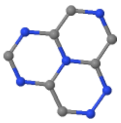	1.9266	1.8288	0.0979	0.2640	-0.1780
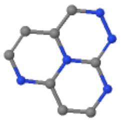	1.7253	1.6803	0.0451	0.2822	-0.1468
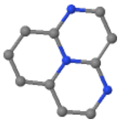	1.8872	1.8739	0.0134	0.1892	-0.3371

Continued on next page

Table 4 – continued from previous page

System	ΔE_{S_1}	ΔE_{T_1}	ΔE_{ST}^{EOM}	ΔE_{ST}^{0}	$\Delta E_{ST}^{sp_\pi}$
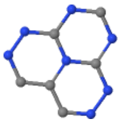	1.8498	1.6613	0.1885	0.4647	0.2810
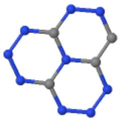	2.4226	2.2764	0.1461	0.3142	-0.1080
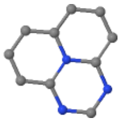	1.7040	1.7943	-0.0902	0.1884	-0.3761
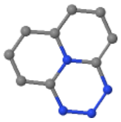	1.4790	1.5001	-0.0210	0.2066	-0.3393
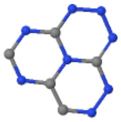	2.4307	2.4021	0.0286	0.2773	-0.2049
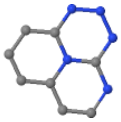	1.7927	1.8311	-0.0384	0.2298	-0.2842
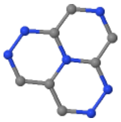	1.1336	1.0111	0.1225	0.2677	-0.1414
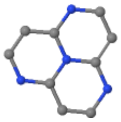	2.2639	2.2448	0.0190	0.1957	-0.3173
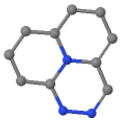	1.6951	1.4436	0.2515	0.2382	-0.1719
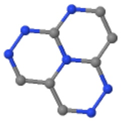	1.6849	1.4434	0.2415	0.3894	0.1484
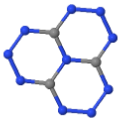	2.0570	2.2065	-0.1495	0.2620	-0.3169

Continued on next page

Table 4 – continued from previous page

System	ΔE_{S_1}	ΔE_{T_1}	$\Delta \mathrm{E}_{ST}^{EOM}$	$\Delta \mathrm{E}_{ST}^{0}$	$\Delta \mathrm{E}_{ST}^{sp_\pi}$
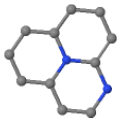	1.4863	1.5203	-0.0340	0.1662	-0.3909
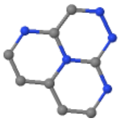	1.9983	1.9428	0.0555	0.2210	-0.2666
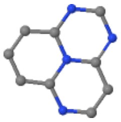	1.9945	2.1101	-0.1156	0.1731	-0.4247
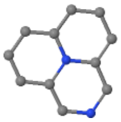	0.9609	0.9673	-0.0064	0.1786	-0.3505
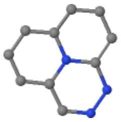	1.6952	1.4437	0.2515	0.2387	-0.1689
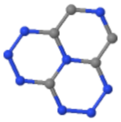	1.6761	1.7203	-0.0441	0.2665	-0.2524
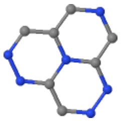	2.4718	1.8863	0.5855	0.3708	0.1679
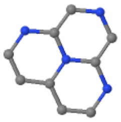	1.5206	1.5837	-0.0632	0.1876	-0.3756
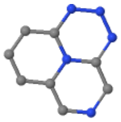	1.3752	1.3727	0.0025	0.2360	-0.2724
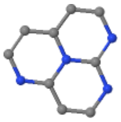	1.9547	2.0403	-0.0857	0.1858	-0.3835
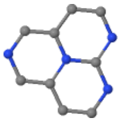	1.5040	1.4417	0.0623	0.3047	-0.0845

Continued on next page

Table 4 – continued from previous page

System	ΔE_{S_1}	ΔE_{T_1}	$\Delta \mathrm{E}_{ST}^{EOM}$	$\Delta \mathrm{E}_{ST}^{0}$	$\Delta \mathrm{E}_{ST}^{sp_\pi}$
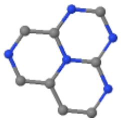	1.8736	1.7874	0.0862	0.3413	-0.0169
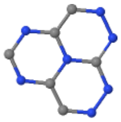	1.8623	1.9206	-0.0584	0.2298	-0.3170
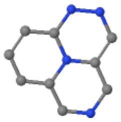	1.8150	1.3883	0.4268	0.3137	0.0311
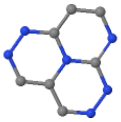	1.6835	1.4424	0.2410	0.3901	0.1497
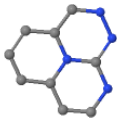	1.6423	1.5592	0.0832	0.2441	-0.2041
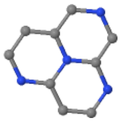	1.8470	1.6839	0.1631	0.2767	-0.1180
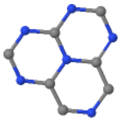	2.1136	2.0697	0.0439	0.3301	-0.0891
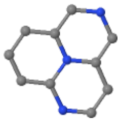	1.4651	1.3270	0.1380	0.2495	-0.1797
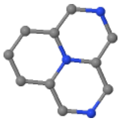	0.8332	0.8073	0.0259	0.1950	-0.3016
